# Clinical impact of the genomic landscape and leukemogenic trajectories in non-intensively treated elderly acute myeloid leukemia patients

**DOI:** 10.1038/s41375-023-01999-6

**Published:** 2023-08-17

**Authors:** Ekaterina Jahn, Maral Saadati, Pierre Fenaux, Marco Gobbi, Gail J. Roboz, Lars Bullinger, Pavlo Lutsik, Anna Riedel, Christoph Plass, Nikolaus Jahn, Claudia Walter, Karlheinz Holzmann, Yong Hao, Sue Naim, Nicholas Schreck, Julia Krzykalla, Axel Benner, Harold N. Keer, Mohammad Azab, Konstanze Döhner, Hartmut Döhner

**Affiliations:** 1 Department of Internal Medicine III, University Hospital of Ulm, Ulm, Germany; 2 Saadati Solutions, Ladenburg, Germany; 3 Hôpital Saint-Louis, Paris, France; 4 Ospedale Policlinico San Martino, Genova, Italy; 5 Weill Cornell Medicine, New York, NY USA; 6 Department of Hematology, Oncology and Cancer Immunology, Charité-Universitätsmedizin Berlin, corporate member of Freie Universität Berlin, Humboldt-Universität zu Berlin, Berlin, Germany; 7 Department of Oncology, Catholic University (KU) Leuven, Leuven, Belgium; 8 Division of Cancer Epigenomics, German Cancer Research Center, Heidelberg, Germany; 9 Genomics Core Facility, Medical Faculty, Ulm University, Ulm, Germany; 10 Astex Pharmaceuticals, Inc., Pleasanton, CA USA; 11 Division of Biostatistics, German Cancer Research Center, Heidelberg, Germany

**Keywords:** Acute myeloid leukaemia, Genetics research, Translational research, Cancer genetics, Genetic testing

## Abstract

To characterize the genomic landscape and leukemogenic pathways of older, newly diagnosed, non-intensively treated patients with AML and to study the clinical implications, comprehensive genetics analyses were performed including targeted DNA sequencing of 263 genes in 604 patients treated in a prospective Phase III clinical trial. Leukemic trajectories were delineated using oncogenetic tree modeling and hierarchical clustering, and prognostic groups were derived from multivariable Cox regression models. Clonal hematopoiesis-related genes (*ASXL1*, *TET2*, *SRSF2*, *DNMT3A*) were most frequently mutated. The oncogenetic modeling algorithm produced a tree with five branches with *ASXL1*, *DDX41*, *DNMT3A*, *TET2*, and *TP53* emanating from the root suggesting leukemia-initiating events which gave rise to further subbranches with distinct subclones. Unsupervised clustering mirrored the genetic groups identified by the tree model. Multivariable analysis identified *FLT3* internal tandem duplications (ITD), *SRSF2*, and *TP53* mutations as poor prognostic factors, while *DDX41* mutations exerted an exceptionally favorable effect. Subsequent backwards elimination based on the Akaike information criterion delineated three genetic risk groups: *DDX41* mutations (favorable-risk), *DDX41*^wildtype^/*FLT3*-ITD^neg^/*TP53*^wildtype^ (intermediate-risk), and *FLT3*-ITD or *TP53* mutations (high-risk). Our data identified distinct trajectories of leukemia development in older AML patients and provide a basis for a clinically meaningful genetic outcome stratification for patients receiving less intensive therapies.

## Introduction

Acute myeloid leukemia (AML) is a disease primarily affecting older individuals with a median age of 68 years at diagnosis [1]. With the advent of the hypomethylating agents (HMA), such as azacitidine and decitabine [2–4], and the HMA-based combination therapies with venetoclax [5, 6] or ivosidenib in *IDH1*-mutated AML [7], there have been significant advances in the therapy of older, unfit patients with AML. Nevertheless, outcome of older individuals remains unsatisfactory due to frequent comorbid conditions and in particular the underlying disease genetics [8].

The genomic landscape of AML has mostly been studied in younger patients who received intensive chemotherapy [9–12]. Data of older patients with newly diagnosed AML receiving less intensive therapies are scarce. However, the available data indicate that the genomic landscape is different from that of younger patients [13–15]. Also, the widely used European LeukemiaNet (ELN) genetic risk classifications [16, 17] have been developed exclusively based on patients who received intensive chemotherapy and may warrant modifications in older patients receiving HMA-based therapies.

The international randomized multi-center phase 3 ASTRAL-1 trial evaluated safety and efficacy of the second-generation HMA guadecitabine (SGI-110) in treatment-naïve AML patients not eligible for intensive chemotherapy in comparison to a treatment choice of either decitabine, azacitidine, or low-dose cytarabine [18]. The trial did not meet its co-primary endpoints of improvement of complete remission rate or of overall survival by guadecitabine in the overall population while ad hoc exploratory analysis favored guadecitabine in patients receiving at least 4 cycles. The ASTRAL-1 trial is the largest study performed in older patients receiving less intensive therapy and offers a unique opportunity to gain insights into the genomic landscape and its clinical impact of AML in older patients.

## Methods

### Patients

In the ASTRAL-1 trial (NCT02348489), 815 patients with previously untreated AML and unfit for intensive chemotherapy were randomly assigned to guadecitabine or treatment choice consisting of azacitidine, decitabine, and low-dose cytarabine [18]. 604 patients gave informed consent for molecular studies and were included in this study; of these patients, *n* = 278 bone marrow and *n* = 326 peripheral blood samples were available. Baseline patient and disease characteristics are given in Table 1. Similar to the overall trial population, there was no difference in outcome by treatment arm in the 604 patients (Supplementary Fig. S1).

**Table 1:** Baseline characteristics of the 604 AML patients.

Female, *n* (%)	255 (42)
Age, median (range), y	77 (59–94)
ECOG performance status, *n* (%) ECOG 0 ECOG 1 ECOG 2 ECOG 3	77 (13) 216 (36) 250 (41) 61 (10)
ELN 2017 risk classification*, *n* (%) Favorable Intermediate Adverse Missing data	101 (17) 124 (21) 363 (62) 16 (3)
ELN 2022 risk classification*, *n* (%) Favorable Intermediate Adverse Missing data	74 (13) 85 (14) 428 (73) 17 (3)
ICC 2022*, *n* (%) AML with myelodysplasia-related gene mutations AML with mutated *TP53* AML with mutated *NPM1* AML not otherwise specified (NOS) AML with myelodysplasia-related cytogenetic abnormalities AML with *MECOM* rearrangements AML with *KMT2A* rearrangements Core-binding factor AML AML with in-frame bZIP mutated *CEBPA* AML with t(9;22)(q34.1;q11.2)/*BCR*::*ABL1* Missing data	266 (45) 102 (17) 93 (16) 60 (10) 30 (5) 17 (3) 9 (1.5) 8 (1.4) 2 ( <1) 1 ( <1) 16 (3)
Therapy-related AML, *n* (%) Missing data, *n*	9 (1.5) 2
AML with myelodysplasia-related changes, *n* (%) Missing data, *n*	162 (27) 2
WBC, median (range) × 10^9^/L	3.6 (0.3–216)
Platelet count, median (range) × 10^9^/L	47 (1–1550)
Hemoglobin, median (range), g/dL	9 (3.5–17)
Peripheral blood blasts [Investigator], median (range) Missing data, *n*	11 (0–99) 0
Peripheral blood blasts [Central], median (range) Missing data, *n*	14 (0–99) 65
Bone marrow blasts [Investigator], median (range) Missing data, *n*	45 (0.8–100) 0
Bone marrow blasts [Central], median (range) Missing data, *n*	59 (1–100) 37
Treatment, *n* (%) Guadecitabine Azacitidine Decitabine Low-dose cytarabine No treatment	290 (48) 154 (25) 124 (21) 21 (3.5) 15 (2.5)
Number of cycles administered, median (range) Guadecitabine Azacitidine Decitabine Low-dose cytarabine	5 (1–38) 6 (1–31) 5 (1–31) 3 (1–18)

Due to small sample sizes, distinct ICC entities are combined into one category, e.g., AML with (8;21)(q22;q22.1)/*RUNX1*::*RUNX1T1*, AML with inv(16)(p13.1q22) and t(16;16)(p13.1;q22)/*CBFB*::*MYH11* was fused to “core-binding factor AML”, AML with inv(3)(q21.3q26.2) or t(3;3)(q21.3;q26.2)/*GATA2, MECOM(EVI1)* or t(3q26.2;v) were fused to “AML with *MECOM* rearrangements”.

*ECOG* Eastern Cooperative Oncology Group, *ELN* European LeukemiaNet, *ICC* International Consensus Classification, *WBC* white blood cell count.

* ELN 2017, ELN 2022, and ICC 2022 were evaluable in 588, 587, and 588 patients, respectively.

Data from conventional cytogenetic analysis and/or fluorescence in-situ hybridization was available for 558 (92%) patients. Data on copy-number variations based on conventional cytogenetics were complemented by data from Illumina HumanMethylation EPIC BeadChip arrays analysis which was performed in 477 patients.

The study was approved by the institutional review board at each participating center. Written consent was given by all patients according to the Declaration of Helsinki.

### Gene mutation analyses

Targeted sequencing (mean read depth: 905x [369–1379]) was performed on the entire coding region of 263 genes involved in hematologic disorders (Supplemental Table S1) using SureSelectXT HS from Agilent Technologies (Santa Clara, CA, USA) for library preparation and a paired-end sequencing (read length: 2 ×100 base pairs) on a HiSeq 2000 platform (Illumina, San Diego, CA, USA). All sequencing data were analyzed using an in-house computational pipeline [19]. The analysis of internal duplications in the *FLT3* gene was performed using GeneScan-based fragment length analysis [20]. All gene mutation analyses were performed centrally at Ulm University Hospital.

### Statistical analyses

The Kaplan–Meier method was used for estimation of survival curves. Differences between survival curves were tested by logrank tests. Mutual exclusivity and co-occurrence of mutations and cytogenetic aberrations with a frequency of ≥ 4% were tested via Fisher’s exact test using a modified Benjamini-Hochberg procedure to control the false discovery rate (FDR) for discrete test statistics [21]. Pairwise associations were measured by odds ratios. Bradley-Terry models were used to assess temporal order of acquisition of mutations (again reduced to those with a frequency of ≥ 4%) based on variant allele frequencies [22]. Oncogenetic trees as proposed by Desper et al. [23] were used to model the dependencies in the sequence of mutation acquisition (for mutations with a frequency of ≥4%) in AML. Trees were developed by an algorithm using non-parametric bootstrap resampling (1000 samples) [24]. To validate the results, a second approach based on maximum likelihood estimation was applied [25]. For clustering observations based on Dirichlet processes, mutations with a frequency of ≥1% were selected. Bayesian Dirichlet processes, which use a mixture model with an infinite prior distribution to model the number and proportion of clusters, were used to classify patients into subgroups in an unsupervised manner [26]. Prognostic factors for overall survival (OS) were identified using a multivariable Cox regression with subsequent backward elimination based on the Akaike information criterion (AIC). The full model included age, sex, ECOG performance status ( ≥ 2 vs.<2), white blood cell counts (log10-transformed), treatment arm, and mutations with a frequency of ≥4%. The results of the reduced model were visualized using predicted survival curves according to Kalbfleisch and Prentice [27] for all combinations of the resulting mutations while clinical variables were fixed at the median or mode. The prediction performance of the full model and the reduced model was compared to a random survival forest (500 trees) based on the same variables and a basic model including the five clinical variables only. The comparisons were based on (integrated) Brier scores using internal validation via 0.632+ bootstrap (*R* = 1000) as implemented in the package pec. All analyses were conducted in R version 4.2.1, packages survival (version 3.3), DiscreteFDR (version 1.3.6), BradleyTerry2 (version 1.1), oncomodel (version 1.0), hdp (version 0.1.5), pec (version 2022.5.4), randomForestSRC(version 3.1.1).

## Results

### Mutational and cytogenetic landscape

**Fig. 1:**
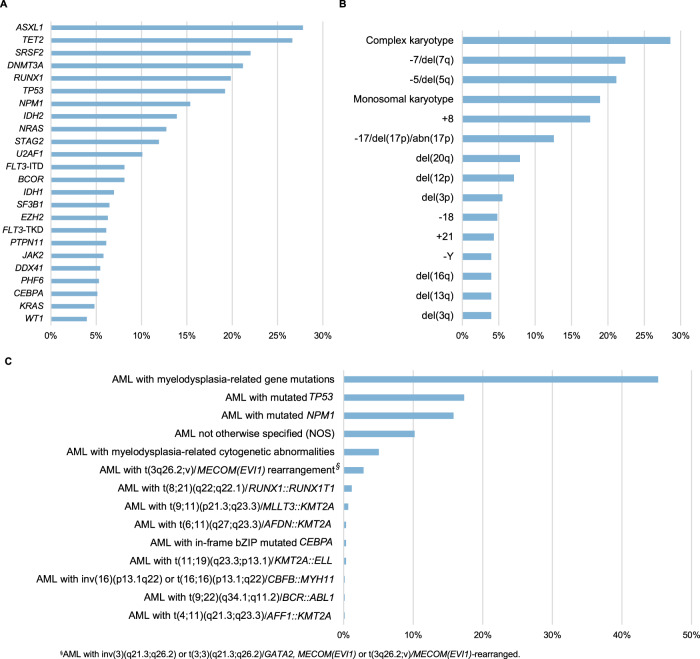
Mutational and cytogenetic landscape of older patients with acute myeloid leukemia. Mutational (**A**) and cytogenetic (**B**) profile, as well as distribution of AML according to the International Consensus Classification (**C**) in 604 older patients with newly diagnosed AML. **A** Genes with mutations present in ≥4% of AML. **B** Cytogenetic abnormalities present in ≥4% of AML; abnormalities were determined by conventional chromosome analysis, fluorescence in-situ hybridization, and EPIC-array analysis. Frequencies given in percent.

In total, *n* = 2985 mutations were detected. The most frequently mutated genes and chromosome abnormalities are shown in Fig. 1A and B and Supplementary Table S2 and S3. Categorization of cases according to the International Consensus Classification of AML [28] is illustrated in Fig. 1C and Supplementary Tables S4 and S5.

Remarkably, a relatively high number of patients with *DDX41* mutations (5.5%, *n* = 33) were identified. Truncating and splice-site mutations with a variant allele frequency (VAF) between 38–60% and thus indicating a germline *DDX41* variant were detected in 61% of the *DDX41* mutated cases (*n* = 20/33), representing 3.3% of the entire patient cohort. The most common co-mutation in suspected germline *DDX41* mutated cases was a second somatic *DDX41* mutation (65%, *n* = 13/20; R525H in 8/13 of the cases) with a markedly lower VAF (median 16% [4–36]), followed by co-mutations in *ASXL1* (20%, *n* = 4/20) and *DNMT3A* (15%, *n* = 3/20) (Supplementary Fig. S2). Upon analysis of associations of genetic lesions, *DDX41* displayed mutual exclusivity with *RUNX1, SRSF2, STAG*2 (in trend), and with adverse cytogenetic aberrations (complex karyotype, −5/del(5q)) (Supplementary Fig. S3); compared to AML with wildtype *DDX41*, AML with mutated *DDX41* also had significantly less co-mutations (5 vs. 3; *p* < .001).

### Oncogenetic tree model using NGS mutational data

**Fig. 2:**
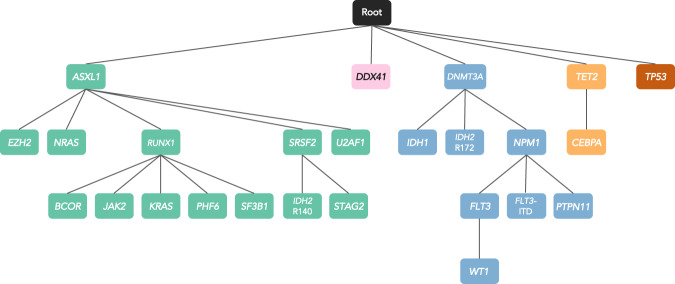
Oncogenetic tree model using a modeling algorithm by Szabo. In an oncogenetic tree model, the root represents a state of disease before occurrence of mutations. Each node represents a gene mutation and each branch represents a distinct biologic clone thus illustrating the different clones and temporal sequence of acquisition of mutations.

To delineate leukemia-initiating trajectories in order to understand whether the leukemogenic in elderly patients differs from that of younger, we constructed an oncogenetic tree by inferring the sequence of mutation acquisition and illustrating the relationships among genetic alterations (Fig. 2) [24].

The algorithm yielded a stable and reproducible oncogenetic tree with five branches with *ASXL1*, *DDX41*, *DNMT3A*, *TET2*, and *TP53* emanating from the root. The data suggest that these genes represent the initiating events which predispose to additional events with further distinct branches.

The tree originating from the *ASXL1* node gave rise to several individual clones with *EZH2*, *NRAS*, *RUNX1*, *SRSF2*, and *U2AF1*; *BCOR, JAK2, KRAS, PHF6*, and *SF3B1* originated from *RUNX1*; and *IDH2*^R140^ and *STAG2* from the *SRSF2* node. Noteworthy, the branches originating from the *ASXL1* node include 8 of the 9 genes defining the ICC category “AML with myelodysplasia-related gene mutations” [28].

In case of the *DNMT3A* node, the oncogenetic tree indicated that the descendent clone will most likely acquire an *NPM1* mutation prior to a *FLT3*-ITD or a *PTPN11* alteration. In general, nodes with mutations in signaling genes (*NRAS, KRAS, JAK2, FLT3, PTPN11*) were located at the very end of each branch, representing last events in driving leukemia, which was also confirmed by a separate analysis using Bradley-Terry models (Supplementary Fig. S4) [19, 22, 29] The *DNMT3A* node produced two other branches with *IDH1* and *IDH2*^R172^ departing separately from the *DNMT3A* node suggesting mutual exclusivity. The *TET2* node generated one branch containing *CEBPA*.

Interestingly, the branches with *DDX41* mutations, which were often germline, and *TP53* terminated at the node without further branching suggesting that mutations in these genes do not depend on or constitute preconditions to further alterations.

### Clustering using mutational and cytogenetic data

**Fig. 3:**
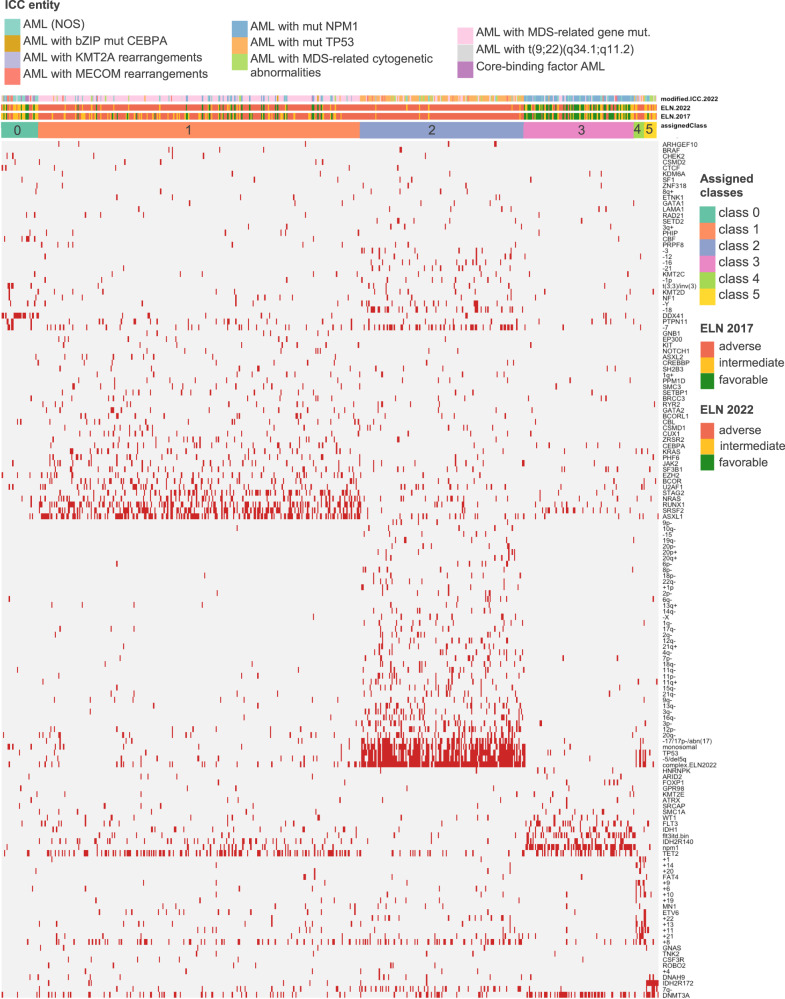
Unsupervised hierarchical clustering using mutational and cytogenetic data. Hierarchical Dirichlet Processes were employed to build the cluster plot. Mutations and cytogenetic data that was present ≥1% of AML were used. The distribution of ELN risk strata and ICC entities across the newly identified classes is indicated by different colors.

To further understand the genetic subgroups in elderly AML patients, now using cytogenetic in addition to molecular data, we performed clustering by hierarchical Dirichlet processes (HDP) which resulted in 5 distinct groups (class 1–5) and an additional group (class 0) whose mutational and cytogenetic data did not lead to a precise classification (Fig. 3).

The largest group (class 1; 49%, *n* = 279) was characterized by the presence of 9 genes defining “AML with myelodysplasia-related gene mutations” and by the presence of chromosomal alterations defining “AML with myelodysplasia-related cytogenetic abnormalities” such as trisomy 8 (17%, *n* = 47), −7/del(7q) (14%, *n* = 39), complex karyotype (9%, *n* = 26), del(20q) (6%, *n* = 16), −5/del(5q) (5%, *n* = 14), del(12p) (3%, *n* = 8), and −17/del(17p)/abn(17p) (3%, *n* = 7). Noteworthy, in this class none of the complex karyotype cases had mutated *TP53*. In comparison to all other classes, class 1 harbored the largest proportion of *NRAS* (73% vs. 27%, *p* < .001) and *KRAS* (79% vs 21%, *p* < .01) mutations.

Class 2 (25%, *n* = 142) consisted mainly of cases with complex karyotype (93%, *n* = 131), −5/del(5q) (72%, *n* = 102), −7/del(7q) (54%, *n* = 77), -17/del(17p)/abn(17p) (44%, *n* = 63), and was strongly associated with *TP53* mutations (70%, *n* = 99); 88% of all *TP53* mutations were associated with class 2.

Class 3 (17%, *n* = 95) was characterized by mutations occurring in de novo AML such as *NPM1* (67%, *n* = 64), *DNMT3A* (48%, *n* = 46), *FLT3*-ITD (35%, *n* = 33), *IDH1* (21%, *n* = 20), and *IDH2*^R140^ (19%, *n* = 18), with virtually no chromosome abnormalities.

Class 4 (*n* = 9 cases only) was characterized by various structural and numerical chromosomal alterations.

Finally, class 5 (*n* = 11) was characterized by *IDH2*^R172^ present in all patients. The most common co-mutation was *DNMT3A* in 73% (*n* = 8).

Upon further analysis of class 0 (6%, *n* = 32), three distinct, non-overlapping subgroups were identified. The first subgroup was defined by *DDX41* alterations, harboring the largest proportion of all *DDX41* mutations (*n* = 18, 58% of all *DDX41*^mut^ in the cluster analysis) and was predominantly associated with a normal karyotype (89%, 16 of 18). The second subgroup harbored 6 of the 8 core-binding-factor leukemia cases. The third group (*n* = 5) was defined by *MECOM*(*EVI1*) rearrangements.

### Current classifications and risk stratifications applied to older AML patients

Based on our observation that leukemia development, the mutational and cytogenetic landscape in elderly AML patients differ from those of younger individuals we aimed to evaluate the informative value of the current classifications and risk stratification in our cohort of older, not-intensively treated population.

#### Overall survival by ELN genetic risk classifications of AML

97% of patients were classified according to 2017 ELN (*n* = 588) and 2022 ELN (*n* = 587) genetic risk classifications [16, 17]. In both schemes, the majority of cases fell into the adverse-risk group (2017 ELN: 62%, *n* = 363; 2022 ELN: 73%, *n* = 428), followed by the intermediate-risk group (2017 ELN: 21%, *n* = 124; 2022 ELN: 14%, *n* = 85), and the favorable-risk group (2017 ELN: 17%, *n* = 101; 2022 ELN: 13%, *n* = 74) (Table 1).

**Fig. 4:**
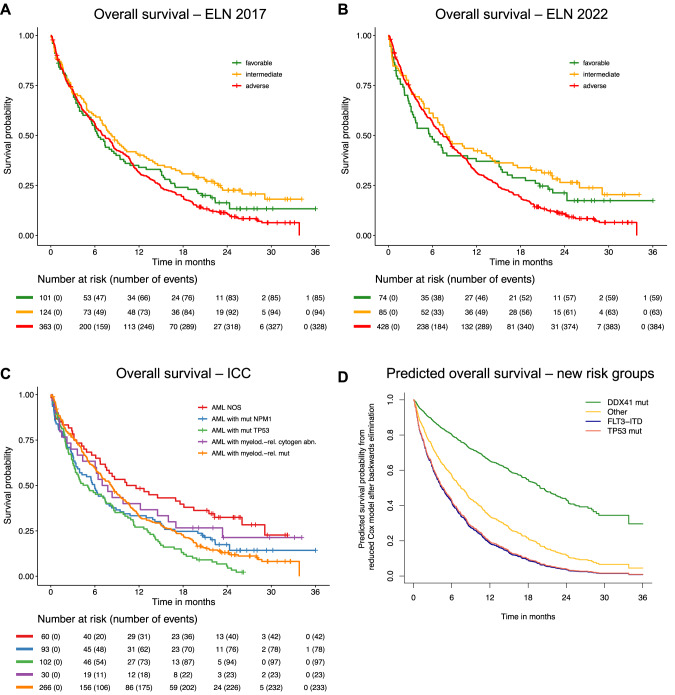
Prognostic value of current AML classifications and of proposed new genetic risk categories for older AML patients. Overall survival by European LeukemiaNet (ELN) 2017 (**A**) and ELN 2022 (**B**) genetic risk classification, by ICC categories (**C**) and proposed risk categories for older AML patients who are not eligible for intensive chemotherapy derived from multivariable Cox models of the current study (**D**). **A, B** Both risk classifications did not provide clinically meaningful separation of the survival curves. In cases previously stratified according to the 2017 ELN stratification, the 2022 ELN stratification entailed a change of strata in 14% (*n* = 82) of the patients, with re-classification to a more adverse-risk category in 13% (*n* = 75) and to a more favorable in 1% (*n* = 6) of the cases. **D** After applying a backwards elimination algorithm on the multivariable Cox model, a reduced prognostic model yielded genetic factors with a significant impact on OS: *DDX41*, *FLT3*-ITD, and *TP53*. The results of the reduced model were visualized using predicted survival probabilities for all combinations of the resulting mutations while clinical variables are fixed at the median or mode. This led to a stratification into three risk categories: *DDX41*^mut^ as favorable, *DDX41*^wt^/*TP53*^wt^/*FLT3*-ITD^neg^ as intermediate, and *TP53*^mut^ or *FLT3*-ITD^pos^ as adverse.

Regarding prognostic value, both risk classifications did not provide clinically meaningful separation of the survival curves (Fig. 4A and B). In both, the adverse-risk group did worst, however, the intermediate- and favorable-risk curves were largely overlapping.

#### Overall survival according to the ICC categories of AML

The generated survival curves did not show a clear separation especially between AML with mutated *NPM1*, AML with myelodysplasia-related gene mutations, and AML with myelodysplasia-related cytogenetic abnormalities (Fig. 4C). With regards to overall survival, patients with “AML with mutated *TP53*” had a dismal prognosis with a 2-year OS of 6%, followed by “AML with myelodysplasia-related gene mutations”, “AML with mutated *NPM1*”, “AML with myelodysplasia-related cytogenetic abnormalities” with 2-year OS rates of 13%, 18%, 21%, respectively (Supplementary Table S6). “AML not otherwise specified (NOS)” were associated with a comparatively favorable prognosis with a 2-year OS rate of 32%. The most commonly mutated gene in “AML NOS” was *DDX41* in 27% (16/60) of the cases and accounted for 49% (16/33) of all *DDX41* mutated cases (Supplementary Table S4). The second largest portion with *n* = 10 of all *n* = 33 *DDX41* mutated cases were found in the category “AML with myelodysplasia-related gene mutations”. To evaluate the prognostic impact of *DDX41* mutations within those ICC subgroups we built Kaplan–Meier estimates (Supplementary Fig. S5). Mutated *DDX41* improved the survival in “AML NOS” and was also associated with improved outcome within the category of “AML with myelodysplasia-related gene mutations”.

### Association of genetic landscape with outcome

In order to address the limitations of the current risk stratifications and classifications in predicting prognosis we assessed whether the genetic markers detected in our study exert an impact on survival. Furthermore, we sought out to identify a new genetic risk classification that is better suited for older, non-intensively treated AML patients.

#### Impact of mutational and cytogenetic features on OS

**Fig. 5:**
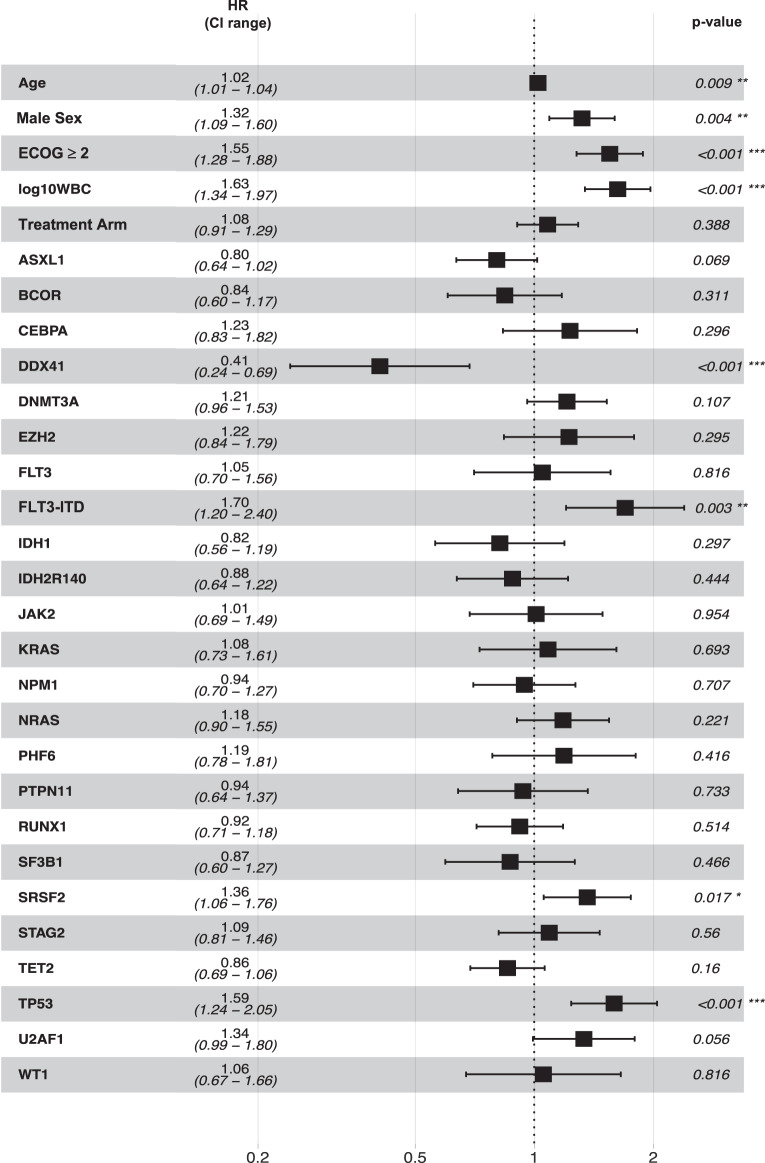
Impact of clinical, mutational, and cytogenetic features on overall survival. Forest plot displaying hazard ratios based on results from Cox regression analysis using clinical variables [age, sex, ECOG performance status ( ≥ 2 vs. <2), white blood cell counts (log_10_-transformed)], treatment, and all mutations with a frequency of ≥4%; HR hazard ratio, CI confidence interval.

In a multivariable Cox model including known clinical prognostic factors, treatment arm, and all gene mutations (“full model”), all clinical variables except treatment arm (Fig. 5) showed a significant detrimental effect on OS (increasing age, male sex, higher ECOG status, increasing WBC). Mutations with an adverse effect were *TP53, FLT3*-ITD, *SRSF2*, and in trend *U2AF1*. In contrast, *DDX41* showed a highly beneficial effect on OS (HR 0.41; 0.24–0.69); and for *ASXL1*, a trend towards longer OS was observed.

A second model (Supplementary Fig. S6) with addition of cytogenetic abnormalities, yielded very similar results with the difference that instead of *TP53* mutations “complex karyotype” was found as a highly significant adverse factor, which may be explained by the strong interaction between the two genetic variables (Supplementary Fig. S7).

OS curves, survival times for selected gene mutations and chromosome abnormalities are given in the Supplement (Supplementary Figs. S8 and S9; Supplemental Tables S7–S10).

#### Identification of biologically relevant mutations for survival outcome

After applying backward elimination on this model, WBC and ECOG status as well as *DDX41*, *FLT3*-ITD and *TP53* remained as prognostic variables in the Cox model for OS. To demonstrate the estimated effects of these variables, the survival probabilities evolving from this model for an exemplary patient with median WBC (3.6 × 10^9^/L) and an ECOG status <2 are depicted in Fig. 4d conditional on the mutational status of *DDX41*, *FLT3*-ITD and *TP53*. Figure 4D is not to be interpreted as a Kaplan–Meier curve as it does not depict estimated survival probabilities for the mutation groups, but rather as predicted survival curves resulting from the multivariable Cox model. Notably, *DDX41*, *TP53* mutations, and *FLT3*-ITD virtually never co-occurred in a patient (*DDX41*/*FLT3*-ITD, *n* = 0; *TP53*/*FLT3*-ITD, *n* = 2; *TP53*/*DDX41*, *n* = 2). The curves show that AML with mutated *DDX41* have a markedly favorable OS, while AML with *FLT3*-ITD or with mutated *TP53* have a similar adverse impact on OS. AML without *DDX41* and *TP5*3 mutations and without the presence of *FLT3*-ITD are associated with an intermediate prognosis.

Although the predicted survival curves show a clear separation, the full model as well as the reduced model yield no improvement in terms of the prediction error compared to a model including the clinical variables only (Supplementary Fig. S10). A model that was able to show better prediction performance compared to the simple clinical model was a random survival forest based on the same variables as the full model. However, predictions from a random forest are gained by averaging the predictions from the single trees, which can be complex models themselves. Hence, it is not possible to find a simple representation of the random forest, which can be used to define new clinically relevant risk groups without losing prediction accuracy.

Nevertheless, we argue that the mutations indicating a prognostic effect are biologically meaningful in the context of AML in elderly patients and even though the model shows no improvement in prediction performance, it does serve as hypothesis generating in terms of future investigations.

## Discussion

This large study enabled us to identify the mutational landscape, to derive distinct disease trajectories and to describe the clinical implications in older, unfit patients with newly diagnosed AML. One important strength of the study is that the data were derived from a single large prospective clinical trial of patients largely treated with an HMA.

In line with previous small studies in older AML patients [13–15], the genetic profile largely differed from that of younger patients, with a predominance of mutations in genes involved in CHIP (*ASXL1*, *TET2*, *SRSF2*, and *DNMT3A*) (Fig. 1A–C), and of adverse-risk molecular and cytogenetic features accounting for 73% by the 2022 ELN risk classification (Fig. 1A–C, Table 1) [17].

CHIP mutations are considered a marker of aging as they accumulate throughout life in individuals without a hematologic phenotype [30–33]. The high prevalence of CHIP mutations in this cohort implies that leukemia of the elderly frequently arises from clonal hematopoiesis, which is supported by our Bradley-Terry model reconstructing the order of temporal acquisition of mutations (Supplementary Fig. S4) [22].

To further analyze leukemia-initiating trajectories, we applied an oncogenetic tree modeling algorithm inferring the sequence of mutation acquisition [24] which produced 5 branches pinpointing to distinct founding clones (Fig. 2). Of note, the *ASXL1* node contained 8 of the 9 genes defining “AML with myelodysplasia-related gene mutations” and 7 of the 8 genes previously reported to define AML with antecedent MDS or myeloproliferative neoplasm [28, 34], providing further evidence for this category as a distinct AML subset. The *DNMT3A* node gave rise to subbranches defined by *NPM1*, *IDH1, IDH2*^R172^ mutations. This temporal sequence is in line with previous observations that preleukemic *DNMT3A* mutations precede the acquisition of leukemia-driving mutations and may persist in remission after intensive chemotherapy [34–37]. Further founding clones encompassed *TP53* and *DDX41* mutations that did not give rise to further subclones, on the one hand providing evidence that these mutations define distinct entities, on the other hand indicating that these leukemia-initiating events are dependent on less transformative events. In fact, both AML with *TP53* and *DDX41* mutations had significantly less co-mutations compared to AML with wildtype *TP53* and *DDX41*, a finding consistent with previous observations [38–41]. HDP clustering of genetic changes aiming at identifying distinct biologic subgroups largely recapitulated our oncogenetic tree model and provided further support for the recently published ICC categories.

Mutations of *DDX41* have recently been recognized as one of the most common predisposition genes for hereditary AML/MDS syndromes and occurring predominantly in older male patients [39–42]. In our cohort, 5.5% of patients exhibited a *DDX41* mutation, with about two-thirds of cases predicted to be of germline origin, which is in line with previous reports in younger patients [39–42]. Overall, the available data, i.e., the findings of *DDX41* mutations being of frequent germline origin, representing leukemia-initiating events, and the paucity of co-occurring molecular and cytogenetic lesions, provide strong evidence that AML with mutated *DDX41* may represent a clinico-pathologic entity of AML.

With regard to the clinical impact of the genomic landscape, both 2017 and 2022 ELN risk stratifications failed to identify clinically meaningful risk groups in this cohort of older patients who received less intensive therapy (Fig. 4A,B). A recent retrospective analysis within the VIALE-A [6] and the preceding phase 1 trial [5] also indicated that ELN risk groups are not prognostic in patients treated with azacitidine plus venetoclax [43].

To identify clinically relevant prognostic factors, we performed multivariable Cox regression analysis for OS including clinical variables and gene mutations. The clinical variables age, sex, and in particular ECOG performance status and WBC retained a strong prognostic impact. Among gene mutations, *DDX41* mutations were identified as a highly prognostic favorable marker, whereas *FLT3*-ITD, *SRSF2*, and in particular *TP53* mutations were unfavorable prognostic factors (Fig. 5). In the reduced model, ECOG performance status, WBC, as well as *DDX41*, *FLT3*-ITD, and *TP53* mutations remained as the most relevant prognostic factors. Figure 4c shows a representation of the model in which three genetically defined risk groups are illustrated by predicted survival curves that are derived from the multivariable model, a favorable-risk group defined by *DDX41* mutation, an adverse-risk groups defined by *FLT3*-ITD or *TP53* mutations, while the remaining patients determine the intermediate-risk group.

An association of *DDX41* mutations with a favorable outcome in AML has recently been described, although in younger intensively treated patients [39–42]. Similarly, in MDS patients mutated *DDX41* has been shown to be associated with a favorable OS after hypomethylating agents, despite being a predictor of AML transformation [44]. In contrast to the data in younger patients, *NPM1* mutation was not associated with a favorable prognosis (Fig. 4C, Supplementary Fig. S8), which in part may be related to a different co-mutation pattern found in older AML patients, e.g., almost half of *NPM1*-mutated AML (45%) had co-occurring myelodysplasia-related gene mutations, most frequently in *SRSF2* and *ASXL1* (Supplementary Fig. S11). The same is true for *NRAS* and *KRAS* mutations that have been suggested to confer resistance to less intense treatment regimens, however did not provide independent prognostic information in our multivariable analysis [14, 43, 45, 46].

Although HMA monotherapy is no longer standard of care for older, unfit patients, similar prognostic factors appear to be relevant for patients treated with azacitidine plus venetoclax, thus, our model may retain its validity also for these patients. In retrospective *post-hoc* analyses of the VIALE-A trial, both AML with *FLT3*-ITD and mutated *TP53* have been shown to be associated with unfavorable outcome even when treated with the combination [47, 48]. *TP53*^multihit^ and *FLT3* mutations have also been identified as top genetic predictors of adverse outcomes in the Molecular International Prognostic Scoring System for Myelodysplastic Syndromes [44].

Outcome data for azacitidine/venetoclax-treated patients exhibiting a *DDX41* mutation are not yet available. Data from a recent retrospective analysis suggest *DDX41* mutation might be associated with effectiveness of monotherapy with HMA [49]. Thus, based on available data, one can assume that these patients will belong to a group of high benefit and long survival times also when treated with the azacitidine and venetoclax.

In conclusion, the data from our study provide unprecedented insights into the genomic landscape of AML in older patients. We identified distinct trajectories of leukemia development, providing support for the new ICC AML disease categories as well as for *DDX41* mutations defining a new clinico-pathologic entity of AML. Our proposed genetic risk model will need to be validated in independent data sets to evaluate whether it may be applicable more broadly also to doublet and triplet HMA-based combination therapies, including targeted agents which are currently in clinical development for patients ineligible for intensive chemotherapy [50].

### Supplementary information

Supplementary Data

## Data Availability

The datasets generated during and/or analyzed during the current study are available from the corresponding author on reasonable request.

## References

[1] SEER, Cancer Stat Facts: acute myeloid leukemia. Bethesda, MD: National Cancer Institute. https://seer.cancer.gov/statfacts/html/amyl.html.

[2] Kantarjian HM, Thomas XG, Dmoszynska A, Wierzbowska A, Mazur G, Mayer J (2012). Multicenter, randomized, open-label, phase III trial of decitabine versus patient choice, with physician advice, of either supportive care or low-dose cytarabine for the treatment of older patients with newly diagnosed acute myeloid leukemia. J Clin Oncol.

[3] Dombret H, Seymour JF, Butrym A, Wierzbowska A, Selleslag D, Jang JH (2015). International phase 3 study of azacitidine vs conventional care regimens in older patients with newly diagnosed AML with >30% blasts. Blood.

[4] Zeidan AM, Fenaux P, Gobbi M, Mayer J, Roboz GJ, Krauter J (2022). Prospective comparison of outcomes with azacitidine and decitabine in patients with AML ineligible for intensive chemotherapy. Blood.

[5] DiNardo CD, Pratz K, Pullarkat V, Jonas BA, Arellano M, Becker PS (2019). Venetoclax combined with decitabine or azacitidine in treatment-naive, elderly patients with acute myeloid leukemia. Blood.

[6] DiNardo CD, Jonas BA, Pullarkat V, Thirman MJ, Garcia JS, Wei AH (2020). Azacitidine and venetoclax in previously untreated acute myeloid leukemia. N Engl J Med.

[7] Montesinos P, Recher C, Vives S, Zarzycka E, Wang J, Bertani G (2022). Ivosidenib and azacitidine in IDH1-mutated acute myeloid leukemia. N Engl J Med.

[8] Döhner H, Weisdorf DJ, Bloomfield CD (2015). Acute myeloid leukemia. N Engl J Med.

[9] Ley TJ, Miller C, Ding L, Raphael BJ, Mungall AJ, Robertson AG (2013). Genomic and epigenomic landscapes of adult de novo acute myeloid leukemia. N Engl J Med.

[10] Papaemmanuil E, Gerstung M, Bullinger L, Gaidzik VI, Paschka P, Roberts ND (2016). Genomic classification and prognosis in acute myeloid leukemia. N Engl J Med.

[11] Bullinger L, Döhner K, Döhner H (2017). Genomics of acute myeloid leukemia diagnosis and pathways. J Clin Oncol.

[12] Tazi Y, Arango-Ossa JE, Zhou Y, Bernard E, Thomas I, Gilkes A (2022). Unified classification and risk-stratification in acute myeloid leukemia. Nat Commun.

[13] Silva P, Neumann M, Schroeder MP, Vosberg S, Schlee C, Isaakidis K (2017). Acute myeloid leukemia in the elderly is characterized by a distinct genetic and epigenetic landscape. Leukemia..

[14] Döhner H, Dolnik A, Tang L, Seymour JF, Minden MD, Stone RM (2018). Cytogenetics and gene mutations influence survival in older patients with acute myeloid leukemia treated with azacitidine or conventional care. Leukemia.

[15] Prassek V, Rothenberg-Thurley M, Sauerland MC, Herold T, Janke H, Ksienzyk B (2018). Genetics of acute myeloid leukemia in the elderly: mutation spectrum and clinical impact in intensively treated patients aged 75 years or older. Haematologica.

[16] Döhner H, Estey E, Grimwade D, Amadori S, Appelbaum FR, Büchner T (2017). Diagnosis and management of AML in adults: 2017 ELN recommendations from an international expert panel. Blood.

[17] Döhner H, Wei AH, Appelbaum FR, Craddock C, DiNardo CD, Dombret H (2022). Diagnosis and management of AML in adults: 2022 recommendations from an international expert panel on behalf of the ELN. Blood.

[18] Fenaux P, Gobbi M, Kropf P, Issa J-PJ, Roboz GJ, Mayer J et al. Guadecitabine vs treatment choice in newly diagnosed acute myeloid leukemia: a global phase 3 randomized study. Blood Adv. 2023;2023010179. 10.1182/bloodadvances.2023010179 PMC10471926 37276510

[19] Jahn N, Terzer T, Sträng E, Dolnik A, Cocciardi S, Panina E (2020). Genomic heterogeneity in core-binding factor acute myeloid leukemia and its clinical implication. Blood Adv.

[20] Stone RM, Mandrekar SJ, Sanford BL, Laumann K, Geyer S, Bloomfield CD (2017). Midostaurin plus chemotherapy for acute myeloid leukemia with a FLT3 mutation. N Engl J Med.

[21] Döhler S, Durand G, Roquain E (2018). New FDR bounds for discrete and heterogeneous tests. Electron J Stat.

[22] Augustin T (2004). Bradley-Terry-Luce models to incorporate within-pair order effects: representation and uniqueness theorems. Br J Math Stat Psychol.

[23] Desper R, Jiang F, Kallioniemi O-P, Moch H, Papadimitriou CH, Schäffer AA (2000). Distance-based reconstruction of Tree Models for oncogenesis. J Comput Biol.

[24] Szabo A, Boucher KM. ONCOGENETIC TREES. In: Handbook of Cancer Models with Applications. World Scientific Publishing Co. Pte. Ltd., Singapore, 2008. pp 1–24.

[25] Heydebreck A, von, Gunawan B, Füzesi L (2004). Maximum likelihood estimation of oncogenetic tree models. Biostatistics.

[26] Teh YW, Jordan MI, Beal MJ, Blei DM (2006). Hierarchical dirichlet processes. J Am Stat Assoc.

[27] Kalbfleisch JD, Prentice RL. The statistical analysis of failure time data. New York: John Wiley & Sons; 1980.

[28] Arber DA, Orazi A, Hasserjian RP, Borowitz MJ, Calvo KR, Kvasnicka H-M (2022). International consensus classification of myeloid neoplasms and acute leukemias: integrating morphologic, clinical, and genomic data. Blood.

[29] Benard BA, Leak LB, Azizi A, Thomas D, Gentles AJ, Majeti R (2021). Clonal architecture predicts clinical outcomes and drug sensitivity in acute myeloid leukemia. Nat Commun.

[30] Park SJ, Bejar R (2018). Clonal hematopoiesis in aging. Curr Stem Cell Rep.

[31] DeZern AE, Malcovati L, Ebert BL. CHIP, CCUS, and other acronyms: definition, implications, and impact on practice. Am Soc Clin Oncol Educ Book. 2019;39:400–10. 10.1200/EDBK_239083 31099654

[32] Jaiswal S, Ebert BL (2019). Clonal hematopoiesis in human aging and disease. Science.

[33] Gondek LP (2021). CHIP: is clonal hematopoiesis a surrogate for aging and other disease?. Hematology.

[34] Lindsley RC, Mar BG, Mazzola E, Grauman PV, Shareef S, Allen SL (2015). Acute myeloid leukemia ontogeny is defined by distinct somatic mutations. Blood.

[35] Shlush LI, Zandi S, Mitchell A, Chen WC, Brandwein JM, Gupta V (2014). Identification of pre-leukaemic haematopoietic stem cells in acute leukaemia. Nature.

[36] Corces-Zimmerman MR, Hong W-J, Weissman IL, Medeiros BC, Majeti R (2014). Preleukemic mutations in human acute myeloid leukemia affect epigenetic regulators and persist in remission. Proc Natl Acad Sci USA.

[37] Gaidzik VI, Weber D, Paschka P, Kaumanns A, Krieger S, Corbacioglu A (2018). DNMT3A mutant transcript levels persist in remission and do not predict outcome in patients with acute myeloid leukemia. Leukemia.

[38] Grob T, Al Hinai ASA, Sanders MA, Kavelaars FG, Rijken M, Gradowska PL (2022). Molecular characterization of mutant TP53 acute myeloid leukemia and high-risk myelodysplastic syndrome. Blood.

[39] Alkhateeb HB, Nanaa A, Viswanatha D, Foran JM, Badar T, Sproat L (2022). Genetic features and clinical outcomes of patients with isolated and comutated DDX41-mutated myeloid neoplasms. Blood Adv.

[40] Duployez N, Largeaud L, Duchmann M, Kim R, Rieunier J, Lambert J (2022). Prognostic impact of DDX41 germline mutations in intensively treated acute myeloid leukemia patients: an ALFA-FILO study. Blood.

[41] Li P, Brown S, Williams M, White T, Xie W, Cui W (2022). The genetic landscape of germline DDX41 variants predisposing to myeloid neoplasms. Blood.

[42] Sébert M, Passet M, Raimbault A, Rahmé R, Raffoux E, Sicre de Fontbrune F (2019). Germline DDX41 mutations define a significant entity within adult MDS/AML patients. Blood.

[43] Döhner H, Pratz KW, DiNardo CD, Jonas BA, Pullarkat VA, Thirman MJ (2022). ELN risk stratification is not predictive of outcomes for treatment-naïve patients with acute myeloid leukemia treated with venetoclax and azacitidine. Blood.

[44] Bernard E, Tuechler H, Greenberg PL, Hasserjian RP, Ossa JEA, Nannya Y (2022). Molecular international prognostic scoring system for myelodysplastic syndromes. NEJM Evid.

[45] Ilyas R, Johnson IM, McCullough K, Al-Kali A, Alkhateeb HB, Begna K (2022). Outcome of patients with acute myeloid leukemia following failure of front-line venetoclax plus hypomethylating agent therapy. Blood.

[46] DiNardo CD, Tiong IS, Quaglieri A, MacRaild S, Loghavi S, Brown FC (2020). Molecular patterns of response and treatment failure after frontline venetoclax combinations in older patients with AML. Blood.

[47] Pollyea DA, Pratz KW, Wei AH, Pullarkat V, Jonas BA, Recher C (2022). Outcomes in patients with poor-risk cytogenetics with or without TP53 mutations treated with venetoclax and azacitidine. Clin Cancer Res.

[48] Konopleva M, Thirman MJ, Pratz KW, Garcia JS, Recher C, Pullarkat V (2022). Impact of FLT3 mutation on outcomes after venetoclax and azacitidine for patients with treatment-naïve acute myeloid leukemia. Clin Cancer Res.

[49] Makishima H, Saiki R, Nannya Y, Korotev S, Gurnari C, Takeda J (2023). Germ line DDX41 mutations define a unique subtype of myeloid neoplasms. Blood.

[50] Döhner H, Wei AH, Löwenberg B (2021). Towards precision medicine for AML. Nat Rev Clin Oncol.

