# Correction: Clinical impact of the genomic landscape and leukemogenic trajectories in non-intensively treated elderly acute myeloid leukemia patients

**DOI:** 10.1038/s41375-023-02017-5

**Published:** 2023-10-03

**Authors:** Ekaterina Jahn, Maral Saadati, Pierre Fenaux, Marco Gobbi, Gail J. Roboz, Lars Bullinger, Pavlo Lutsik, Anna Riedel, Christoph Plass, Nikolaus Jahn, Claudia Walter, Karlheinz Holzmann, Yong Hao, Sue Naim, Nicholas Schreck, Julia Krzykalla, Axel Benner, Harold N. Keer, Mohammad Azab, Konstanze Döhner, Hartmut Döhner

**Affiliations:** 1 Department of Internal Medicine III, University Hospital of Ulm, Ulm, Germany; 2 Saadati Solutions, Ladenburg, Germany; 3 Hôpital Saint-Louis, Paris, France; 4 Ospedale Policlinico San Martino, Genova, Italy; 5 Weill Cornell Medicine, New York, NY USA; 6 Department of Hematology, Oncology and Cancer Immunology, Charité-Universitätsmedizin Berlin, Corporate Member of Freie Universität Berlin, Humboldt-Universität zu Berlin, Berlin, Germany; 7 Department of Oncology, Catholic University (KU) Leuven, Leuven, Belgium; 8 Division of Cancer Epigenomics, German Cancer Research Center, Heidelberg, Germany; 9 Genomics Core Facility, Medical Faculty, Ulm University, Ulm, Germany; 10 Astex Pharmaceuticals, Inc., Pleasanton, CA USA; 11 Division of Biostatistics, German Cancer Research Center, Heidelberg, Germany

**Keywords:** Acute myeloid leukaemia, Genetics research, Translational research, Cancer genetics, Genetic testing

Correction to: *Leukemia* 10.1038/s41375-023-01999-6, published online 17 August 2023

During the correction process, Table 1 was incorrectly formatted by the typesetter. The assignment of the right column did not match the information in the left column.

**Table 1:** Baseline characteristics of the 604 AML patients.

Female, *n* (%)	255 (42)
Age, median (range), y	77 (59–94)
ECOG performance status, *n* (%) ECOG 0 ECOG 1 ECOG 2 ECOG 3	77 (13) 216 (36) 250 (41) 61 (10)
ELN 2017 risk classification*, *n* (%) Favorable Intermediate Adverse Missing data	101 (17) 124 (21) 363 (62) 16 (3)
ELN 2022 risk classification*, *n* (%) Favorable Intermediate Adverse Missing data	74 (13) 85 (14) 428 (73) 17 (3)
ICC 2022*, *n* (%) AML with myelodysplasia-related gene mutations AML with mutated *TP53* AML with mutated *NPM1* AML not otherwise specified (NOS) AML with myelodysplasia-related cytogenetic abnormalities AML with *MECOM* rearrangements AML with *KMT2A* rearrangements Core-binding factor AML AML with in-frame bZIP mutated *CEBPA* AML with t(9;22)(q34.1;q11.2)/*BCR*::*ABL1* Missing data	266 (45) 102 (17) 93 (16) 60 (10) 30 (5) 17 (3) 9 (1.5) 8 (1.4) 2 ( <1) 1 ( <1) 16 (3)
Therapy-related AML, *n* (%) Missing data, *n*	9 (1.5) 2
AML with myelodysplasia-related changes, *n* (%) Missing data, *n*	162 (27) 2
WBC, median (range) × 10^9^/L	3.6 (0.3–216)
Platelet count, median (range) × 10^9^/L	47 (1–1550)
Hemoglobin, median (range), g/dL	9 (3.5–17)
Peripheral blood blasts [Investigator], median (range) Missing data, *n*	11 (0–99) 0
Peripheral blood blasts [Central], median (range) Missing data, *n*	14 (0–99) 65
Bone marrow blasts [Investigator], median (range) Missing data, *n*	45 (0.8–100) 0
Bone marrow blasts [Central], median (range) Missing data, *n*	59 (1–100) 37
Treatment, *n* (%) Guadecitabine Azacitidine Decitabine Low-dose cytarabine No treatment	290 (48) 154 (25) 124 (21) 21 (3.5) 15 (2.5)
Number of cycles administered, median (range) Guadecitabine Azacitidine Decitabine Low-dose cytarabine	5 (1–38) 6 (1–31) 5 (1–31) 3 (1–18)

Due to small sample sizes, distinct ICC entities are combined into one category, e.g., AML with (8;21)(q22;q22.1)/*RUNX1*::*RUNX1T1*, AML with inv(16)(p13.1q22) and t(16;16)(p13.1;q22)/*CBFB*::*MYH11* was fused to “core-binding factor AML”, AML with inv(3)(q21.3q26.2) or t(3;3)(q21.3;q26.2)/*GATA2, MECOM(EVI1)* or t(3q26.2;v) were fused to “AML with *MECOM* rearrangements”.

*ECOG* Eastern Cooperative Oncology Group, *ELN* European LeukemiaNet, *ICC* International Consensus Classification, *WBC* white blood cell count.

* ELN 2017, ELN 2022, and ICC 2022 were evaluable in 588, 587, and 588 patients, respectively.

The table should have appeared as shown below. The original article has been corrected.

