# Supplementary material for: Clinical impact of the genomic landscape and leukemogenic trajectories in non-intensively treated elderly acute myeloid leukemia patients
Source: Leukemia. 2023 Aug 17;37(11):2187–96. doi: 10.1038/s41375-023-01999-6 (PMC10624608; doi:10.1038/s41375-023-01999-6)
Supplement: Supplementary file 1 — Supplementary Data [file 41375_2023_1999_MOESM1_ESM.docx]

**SUPPLEMENTARY DATA**

**Clinical Impact of the Genomic Landscape and Leukemogenic Trajectories in Non-Intensively Treated Elderly Acute Myeloid Leukemia Patients**

Ekaterina Jahn,^1^ Maral Saadati,^2^ Pierre Fenaux,^3^ Marco Gobbi,^4^ Gail J. Roboz,^5^ Lars Bullinger,^6^ Pavlo Lutsik,^7^ Anna Riedel,^8^ Christoph Plass,^8^ Nikolaus Jahn,^1^ Claudia Walter,^1^ Karlheinz Holzmann,^9^ Yong Hao,^10^ Sue Naim,^10^ Nicholas Schreck,^11^ Julia Krzykalla,^11^ Axel Benner,^11^ Harold N. Keer,^10^ Mohammad Azab,^10^ Konstanze Döhner,^1*^ Hartmut Döhner^1*^

^1^ Department of Internal Medicine III, University Hospital of Ulm, Ulm, Germany

^2^ Saadati Solutions, Ladenburg, Germany

^3^ Hôpital Saint-Louis, Paris, France

^4^ Ospedale Policlinico San Martino, Genova, Italy

^5^ Weill Cornell Medicine, New York, NY

^6^ Department of Hematology, Oncology and Cancer Immunology, Charité-Universitätsmedizin Berlin, corporate member of Freie Universität Berlin, Humboldt-Universität zu Berlin, Berlin, Germany

^7^ Department of Oncology, Catholic University (KU) Leuven, Leuven, Belgium

^8^ Division of Cancer Epigenomics, German Cancer Research Center, Heidelberg, Germany

^9^ Genomics Core Facility, Medical Faculty, Ulm University

^10^ Astex Pharmaceuticals, Inc., Pleasanton, CA, USA

^11^ Division of Biostatistics, German Cancer Research Center, Heidelberg, Germany

* Contributed equally

**SUPPLEMENTARY METHODS**

**Copy number variation (CNV) analysis:**

Copy-number variations were inferred from Illumina HumanMethylation EPIC BeadChip arrays which were measured according to the manufacturer’s protocol. Methylation data was processed using the RnBeads pipeline (http://rnbeads.mpi-inf.mpg.de/) using the default parameters. Probes were excluded from the dataset if they were measuring methylation on a non-CpG context, if they are potentially cross-reactive, if they had more than 10% missing values across samples, if the number of microarray beads was below a threshold of 3. Furthermore, a *greedycut* procedure inside RnBeads was used to remove probes with systematically high detection p-values. Additionally, probes overlapping with common single-nucleotide polymorphisms with minor allele frequency above 0.01 based on dbSNP v.151 were excluded. EPIC array measurements were used for CNA analysis using *conumee* package (Hovestadt, V. & Zapatka, M. conumee: Enhanced copy-number variation analysis using Illumina DNA methylation arrays. R package version 1.9.0 (<http://bioconductor.org/packages/conumee/>) by combining the signal intensities for the methylated and unmethylated channels of each CpG, combining neighboring probes into bins with minimum size of 50 kb and containing least 15 probes and comparing them against respective values from a set of normal control samples. In a first step, samples were analyzed without a control data set. CNV plots were manually inspected and 25 samples with a normal CNV pattern were designated “controls”. In a second step the analysis was repeated using these 25 samples as controls. Again, CNV plots were annotated by visual inspection and gains/losses of chromosome arms were assigned to each sample.

**SUPPLEMENTARY TABLES**

| ABCA12 | CEBPA | DNMT3B | GNB1 | LUC7L2 | NUMA1 | RASA3 | SRP72 |
| --- | --- | --- | --- | --- | --- | --- | --- |
| ABL1 | CHEK2 | DYNC1H1 | H3F3A | MAP3K10 | NUP98 | RASEF | SRSF2 |
| ACIN1 | TERC | EED | H3F3B | MAP3K11 | NXF1 | RASGRF1 | STAG1 |
| ACSS3 | CCDC26 | EEFSEC | HAX1 | MAP3K4 | OBSCN^§^ | RB1 | STAG2 |
| ADGRV1 | CLTCL1 | EGFR | HCN1 | MAP3K9 | OMG | RBBP5 | STAT3 |
| ALK | CNNM2 | ELANE | HIPK2 | MBD4 | PAX5 | RBBP6 | STAT5A |
| ANKRD26 | COPRS | EP300 | HNRNPK | MGA | PDGFB | RBMX | SUZ12 |
| ARHGEF10 | CREBBP | EPHA6 | HRAS | MN1 | PDGFRB | RET | SYNE1^§^ |
| ARID1A | CSF1R | ETNK1 | IDH1 | MPL | PDPK1 | RHOA | TCIRG1 |
| ARID2 | CSF2RB | ETV6 | IDH2 | MST1 | PHF6 | RMI1 | TERT |
| AS3MT | CSF3R | EVI2A | IDNK | MYC | PHIP | ROBO1 | TET1 |
| ASXL1 | CSMD1 | EVI2B | IKZF2 | MYH9 | PHKG1 | ROBO2 | TET2 |
| ASXL2 | CSMD2 | EWSR1 | INPP5D | MYLK2 | PIK3CA | RPS6KA2 | TINF2 |
| ATRX | CSNK1A1 | EZH1 | IRF1 | MYO1F | PKN2 | RPS6KA3 | TNK1 |
| BAP1 | CTC1 | EZH2 | IRF4 | NCOA7 | PLEKHH1 | RPS6KA6 | TNK2 |
| BCL10 | CTCF | FAM175A | IRF8 | NDE1 | PLEKHS1 | RRAS | TP53 |
| BCL2 | CTNNB1 | FAM5C | JAK1 | NEK2 | PPM1D | RUNX1 | TTC39A |
| BCOR | CUX1 | FAT4 | JAK2 | NF1 | PRKAG2 | RYR2 | U2AF1 |
| BCORL1 | DCC | FBXW7 | JAK3 | NF2 | PRKG2 | SAMHD1 | U2AF2 |
| BCR | DDX23 | FGFR2 | JARID2 | NFE2 | PRPF40A | SETBP1 | UBQLN1 |
| BRAF | DDX4 | FLG | KAT6A | NFE2L1 | PRPF40B | SETD2 | UBXN11 |
| BRCC3 | DDX41 | FLT3 | KDM5C | NFE2L2 | PRPF8 | SETDB1 | WAC |
| C6 | DDX54 | FOXP1 | KDM6A | NIPBL | PTEN | SF1 | WHSC1 |
| CALR | DHX15 | FRMD3 | KDR | NOTCH1 | PTPN11 | SF3A1 | WRAP53 |
| CBL | DHX33 | G6PC3 | KIF27 | NOTCH2 | PTPRF | SF3B1 | WT1 |
| CCND1 | DICER1 | GALNT11 | KIT | NPM1 | PTPRT | SH2B3 | YLPM1 |
| CCND2 | DIS3 | GALNTL5 | KMT2A | NRAS | PXDN | SMARCB1 | ZBTB33 |
| CDHR1 | DKC1 | GATA1 | KMT2C | NRXN1 | RAB11FIP4 | SMC1A | ZBTB7A |
| CDK4 | DNAH9 | GATA2 | KMT2D | NRXN3 | RAC1 | SMC3 | ZMYM3 |
| CDKN1B | DNAJB8 | GFI1 | KMT2E | NSD1 | RAD21 | SMG1 | ZNF318 |
| CDKN2A | DND1 | GIGYF2 | KRAS | NT5C2 | RAD50 | SPI1 | ZNF687 |
| CDKN2B | DNM2 | GKAP1 | LAMA1 | NTRK1 | RAD51 | SPRED2 | ZRSR2 |
| CDKN2C | DNMT3A | GNAS | LAMC3 | NTRK3 | RASA2 | SRCAP |  |

**Table S1.** List of all genes (n=263) targeted by custom sequencing panel.

^$^OBSCN and SYNE1 were excluded from the analysis due to numerous false positive results and variants of unknown significance.

**Table S2.** Prevalence of gene mutations. Table displays the number of AML with mutations and not the number of total mutations in the cohort.

**
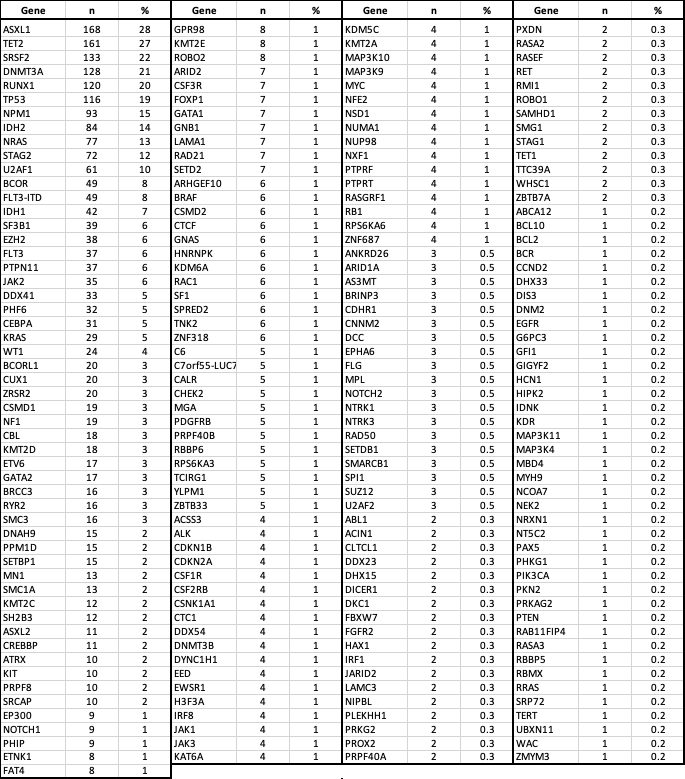
**

**Table S3.** Prevalence of chromosome abnormalities. Table displays the number of AML with respective cytogenetic abnormalities and not the number of total alterations in the cohort.

**
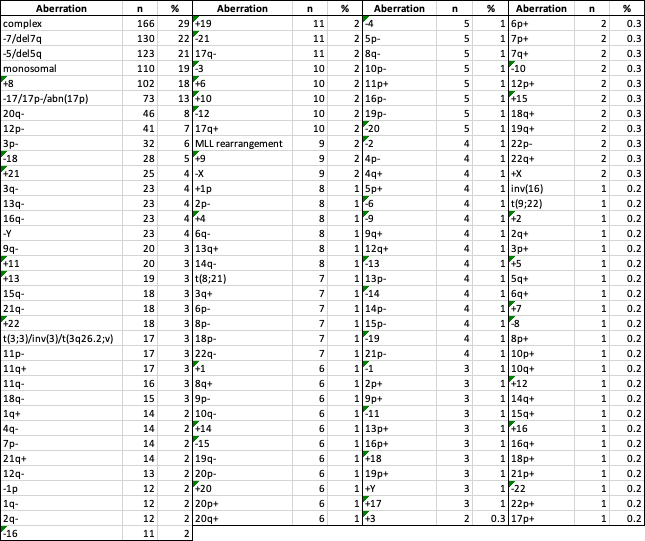
**

**Table S4.** Frequency of mutations by ICC categories. Due to small sample sizes, distinct entities are combined into one category, e.g., AML with (8;21)(q22;q22.1)/*RUNX1::RUNX1T1*, AML with inv(16)(p13.1q22) and t(16;16)(p13.1;q22)/*CBFB::MYH11* was fused to “core-binding factor AML”, AML with inv(3)(q21.3q26.2), t(3;3)(q21.3;q26.2)/*GATA2, MECOM*(*EVI1*), and t(3q26.2;v)/*MECOM*-rearranged were fused to “AML with *MECOM* rearrangements”. Alterations present in ≥5% of AML in each category are displayed in red. Note that only patients with *TP53* mutations with a variant allele frequency of ≥10% were assigned to the group “AML with mutated *TP53*”.

**Table S5.** Frequency of cytogenetic alterations by ICC categories. Due to small sample sizes distinct entities are combined into one category, e.g., AML with (8;21)(q22;q22.1)/*RUNX1*::*RUNX1T1*, AML with inv(16)(p13.1q22) and t(16;16)(p13.1;q22)/*CBFB*::*MYH11* was fused to “core-binding factor AML”, AML with inv(3)(q21.3q26.2), t(3;3)(q21.3;q26.2)/*GATA2*, *MECOM*(*EVI1)*, and t(3q26.2;v)/*MECOM*-rearranged were fused to “AML with *MECOM* rearrangements”. Alterations present in ≥5% of the patients in each category are displayed in red.

**Table S6.** Median overall survival and 1- and 2-year overall survival rates of patients by categories (present in ≥5%) of the International Consensus Classification for AML.

|  | Median OS (months) | Time | Survival (%) | Std. error | 95% CI |
| --- | --- | --- | --- | --- | --- |
| AML not otherwise specified (NOS) | 11.1 | 1-year OS  2-year OS | 0.48  0.32 | 0.065  0.062 | 0.37-0.63  0.22-0.47 |
| AML with myelodysplasia-related gene mutations | 8.3 | 1-year OS  2-year OS | 0.33  0.13 | 0.029  0.021 | 0.28-0.39  0.09-0.18 |
| AML with myelodysplasia-related cytogenetic abnormalities | 7.5 | 1-year OS  2-year OS | 0.40  0.21 | 0.089  0.080 | 0.26-0.62  0.10-0.47 |
| AML with mutated *NPM1* | 6.0 | 1-year OS  2-year OS | 0.33  0.18 | 0.049  0.040 | 0.25-0.44  0.11-0.27 |
| AML with mutated *TP53* | 5.1 | 1-year OS  2-year OS | 0.27  0.06 | 0.044  0.023 | 0.19-0.37  0.03-0.13 |

**Table S7.** One- and 2- year overall survival rates for recurrent mutations and chromosomal abnormalities in AML and/or for mutations and chromosomal abnormalities with known prognostic effect. The corresponding Kaplan-Meier estimates are illustrated in Figure S8 and S9.

| Gene |  | Survival (%) | Std. error | 95% CI |
| --- | --- | --- | --- | --- |
| *DDX41* | 1-year wildtype  mutated  2-year wildtype  mutated | 0.3212  0.7273  0.1196  0.6008 | 0.0197  0.0775  0.0141  0.0861 | 0.2849 - 0.3622  0.5901 - 0.8963  0.0948 - 0.1507  0.4537 - 0.7955 |
| *TP53* | 1-year wildtype  mutated  2-year wildtype  mutated | 0.3605  0.2727  0.1619  0.0735 | 0.0219  0.0173  0.017  0.0254 | 0.3201 - 0.4060  0.2070 - 0.3612  0.1313 - 0.1997  0.0373 - 0.1448 |
| *FLT3-*ITD*^§^* | 1-year negative  positive  2-year wildtype  mutated | 0.3568  0.2041  N/A  N/A | 0.0205  0.0576  N/A  N/A | 0.3188 - 0.399  0.1174 - 0.3548  N/A  N/A |
| *NPM1* | 1-year negative  positive  2-year wildtype  mutated | 0.3455  0.3333  0.139  0.1745 | 0.0212  0.0489  0.016  0.0404 | 0.3064 - 0.3897  0.2501 - 0.4443  0.111 - 0.174  0.1108 - 0.2748 |
| *DNMT3A* | 1-year negative  positive  2-year wildtype  mutated | 0.348  0.3271  0.1477  0.1358 | 0.022  0.0418  0.0169  0.0315 | 0.2546 - 0.4203  0.1174 - 0.3548  0.1181 - 0.1848  0.0861- 0.2140 |
| *TET2* | 1-year negative  positive  2-year wildtype  mutated | 0.364  0.290  0.1422  0.1522 | 0.023  0.036  0.0173  0.0291 | 0.321 - 0.412  0.227 - 0.369  0.1119 - 0.1806  0.1047 - 0.2213 |
| *ASXL1* | 1-year negative  positive  2-year wildtype  mutated | 0.3437  0.3440  0.1468  0.1388 | 0.0229  0.0369  0.0177  0.0277 | 0.3016 - 0.3916  0.2788 - 0.4245  0.1159 - 0.1859  0.0939 - 0.2052 |
| *BCOR* | 1-year negative  positive  2-year wildtype  mutated | 0.3361  0.4286  0.1465  0.1319 | 0.0202  0.0707  0.0156  0.0495 | 0.2988 - 0.3782  0.3102 - 0.5922  0.1189 - 0.1805  0.0632 - 0.2751 |
| *EZH2* | 1-year negative  positive  2-year wildtype  mutated | 0.3450  0.325  0.1469  0.1095 | 0.0201  0.077  0.0154  0.0558 | 0.3078 - 0.3868  0.204 - 0.517  0.1196 - 0.1805  0.0404 - 0.2970 |
| *RUNX1* | 1-year negative  positive  2-year wildtype  mutated | 0.358  0.2858  0.1556  0.1025 | 0.022  0.0414  0.0172  0.0286 | 0.318 - 0.404  0.2151 - 0.3796  0.1254 - 0.1932  0.0593 - 0.1770 |
| *SF3B1* | 1-year negative  positive  2-year wildtype  mutated | 0.3433  0.3500  0.1415  0.187 | 0.0201  0.0754  0.0153  0.064 | 0.3060 - 0.3851  0.2294 - 0.5339  0.1145 - 0.1749  0.096 - 0.366 |
| *SRSF2* | 1-year negative  positive  2-year wildtype  mutated | 0.3637  0.2734  0.1637  0.0836 | 0.0223  0.0388  0.0178  0.0241 | 0.3225 - 0.4103  0.2070 - 0.3612  0.1323 - 0.2026  0.0475 - 0.1471 |
| *STAG2* | 1-year negative  positive  2-year wildtype  mutated | 0.3514  0.2870  0.1489  0.1148 | 0.0208  0.0537  0.0161  0.0381 | 0.3128 - 0.3947  0.1989 - 0.4143  0.1204 - 0.1840  0.0599 - 0.2200 |
| *U2AF1* | 1-year negative  positive  2-year wildtype  mutated | 0.3504  0.2838  0.152  0.0835 | 0.0206  0.0582  0.016  0.0357 | 0.3123 - 0.3932  0.1899 - 0.4244  0.124 - 0.187  0.0361 - 0.1932 |
| *ZRSR2* | 1-year negative  positive  2-year wildtype  mutated | 0.3435  0.350  0.1469  0.0750 | 0.0198  0.107  0.0152  0.0664 | 0.3068 - 0.3845  0.193 - 0.636  0.1198 - 0.1800  0.0132 - 0.4250 |
| *IDH1* | 1-year wildtype  mutated  2-year wildtype  mutated | 0.3419  0.3699  0.1423  0.1758 | 0.0201  0.0755  0.0153  0.0636 | 0.3046 - 0.3837  0.2480 - 0.5518  0.1153 - 0.1756  0.0865 - 0.3573 |
| *IDH2* | 1-year wildtype  mutated  2-year wildtype  mutated  Survival rate by mutated alleles:  1-year *IDH2*R140  *IDH2*R172  2-year *IDH2*R140  *IDH2*R172 | 0.3364  0.391  0.1380  0.1903  0.3890  0.364  0.1556  0.312 | 0.0208  0.054  0.0157  0.0454  0.0638  0.103  0.0486  0.100 | 0.2980 - 0.3798  0.298 - 0.512  0.1104 - 0.1724  0.1193 - 0.3036  0.2820 - 0.5365  0.209 - 0.632  0.0844 - 0.2868  0.166 - 0.585 |
| *NRAS* | 1-year wildtype  mutated  2-year wildtype  mutated | 0.358  0.2468  0.1552  0.0758 | 0.021  0.0491  0.0164  0.0306 | 0.319 - 0.402  0.1670 - 0.3645  0.1261 - 0.1911  0.0343 - 0.1672 |
| *KRAS^§^* | 1-year wildtype  mutated  2-year wildtype  mutated | 0.35  0.24  N/A  N/A | 0.020  0.0795  N/A  N/A | 0.312 - 0.390  0.1266 - 0.4602  N/A  N/A |
| *FLT3-*TKD | 1-year negative  positive  2-year wildtype  mutated | 0.3432  0.3514  0.1423  0.1892 | 0.0201  0.0785  0.0153  0.0644 | 0.3060 - 0.3849  0.2268 - 0.5443  0.1153 - 0.1756  0.0971 - 0.3686 |
| *PTPN11* | 1-year negative  positive  2-year wildtype  mutated | 0.341  0.3906  0.1473  0.1116 | 0.020  0.0814  0.0155  0.0526 | 0.304 - 0.382  0.2597 - 0.5875  0.1198 - 0.1809  0.0443 - 0.2810 |

^§^ 2-year survival could not be calculated due to no subjects with this alteration alive at the 2-year mark.

**Table S8.** One- and 2- year overall survival rates for recurrent mutations and chromosomal abnormalities in AML and/or for mutations and chromosomal abnormalities with known prognostic effect. The corresponding Kaplan-Meier estimates are illustrated in Figure S8 and S9.

| Abnormality |  | Survival (%) | Std. error | 95% CI |
| --- | --- | --- | --- | --- |
| Complex karyotype | 1-year negative  positive  2-year negative  positive | 0.3756  0.2566  0.1808  0.0597 | 0.0236  0.0341  0.0193  0.0196 | 0.3320 - 0.4249  0.1977 - 0.3330  0.1466 - 0.2228  0.0314 - 0.1136 |
| Monosomal karyotype | 1-year negative  positive  2-year negative  positive | 0.3671  0.234  0.1686  0.0525 | 0.0221  0.041  0.0177  0.0225 | 0.3263 - 0.4131  0.166 - 0.330  0.1372 - 0.2070  0.0227 - 0.1215 |
| -5/del5q | 1-year negative  positive  2-year negative  positive | 0.3476  0.3212  0.1626  0.0878 | 0.0221  0.0429  0.0176  0.0267 | 0.3069 - 0.3937  0.2473 - 0.4172  0.1316 - 0.2011  0.0484 - 0.1592 |
| -7/del7q | 1-year negative  positive  2-year negative  positive | 0.3562  0.2919  0.1670  0.0680 | 0.0224  0.0404  0.0178  0.0259 | 0.3149 - 0.4029  0.2226 - 0.3828  0.1354 - 0.2059  0.0322 - 0.1435 |
| +8 | 1-year negative  positive  2-year negative  positive | 0.3599  0.2577  0.1583  0.0919 | 0.0218  0.0435  0.0171  0.0300 | 0.3195 - 0.4053  0.1851 - 0.3589  0.1281 - 0.1956  0.0485 - 0.1741 |
| -17/del(17p)/abn(17p) | 1-year negative  positive  2-year negative  positive | 0.3543  0.2544  0.1584  0.0646 | 0.0211  0.0517  0.0166  0.0302 | 0.3153 - 0.3982  0.1708 - 0.3790  0.1290 - 0.1945  0.0259 - 0.1613 |
| del(20q) | 1-year negative  positive  2-year negative  positive | 0.3514  0.2293  0.1561  0.0287 | 0.0205  0.0636  0.0161  0.0276 | 0.3134 - 0.3941  0.1332 - 0.3948  0.1276 - 0.1910  0.00434 - 0.1890 |
| del(12p)^§^ | 1-year wildtype  mutated  2-year wildtype  mutated | 0.3415  0.3185  N/A  N/A | 0.0203  0.0756  N/A  N/A | 0.3039 - 0.3838  0.2309 - 0.5360  N/A  N/A |
| del3p | 1-year negative  positive  2-year negative  positive | 0.3457  0.2812  0.1522  0.0625 | 0.0202  0.0795  0.0157  0.0428 | 0.3082 - 0.3878  0.1616 - 0.4894  0.1242 - 0.1864  0.0163 - 0.2391 |
| Monosomy 18 | 1-year negative  positive  2-year negative  positive | 0.3490  0.1938  0.1519  0.0388 | 0.0202  0.0777  0.0157  0.0380 | 0.3117 - 0.3909  0.0883 - 0.4254  0.1240 - 0.1859  0.00567 - 0.2650 |

| Gene | Allele | Median OS (Months) |
| --- | --- | --- |
| *DDX41* | wildtype  mutated | 7.1  27.8 |
| *TP53* | wildtype  mutated | 8.0  5.3 |
| *FLT3-*ITD | negative  positive | 8.1  4.9 |
| *NPM1* | wildtype  mutated | 8.2  6.1 |
| *DNMT3A* | wildtype  mutated | 7.6  7.2 |
| *TET2* | wildtype  mutated | 8.0  6.8 |
| *ASXL1* | wildtype  mutated | 7.2  8.7 |
| *BCOR* | wildtype  mutated | 7.5  10.8 |
| *EZH2* | wildtype  mutated | 7.5  9.1 |
| *RUNX1* | wildtype  mutated | 7.5  8 |
| *SF3B1* | wildtype  mutated | 7.5  8.1 |
| *SRSF2* | wildtype  mutated | 7.5  7.1 |
| *STAG2* | wildtype  mutated | 7.5  7.6 |
| *U2AF1* | wildtype  mutated | 7.7  5.6 |
| *ZRSR2* | wildtype  mutated | 7.5  7.0 |
| *IDH1* | wildtype  mutated | 7.5  6.6 |
| *IDH2* | wildtype  mutated  Median OS by mutated alleles:  *IDH2*R140  *IDH2*R172 | 7.5  7.9  8.0  5.0 |
| *NRAS* | wildtype  mutated | 7.5  7.3 |
| *KRAS* | wildtype  mutated | 7.5  5.1 |
| *FLT3-*TKD | negative  positive | 7.5  5.5 |
| *PTPN11* | wildtype  mutated | 7.5  7.5 |

**Table S9.** Median overall survival (OS) times for recurrent mutations abnormalities in AML and/or for mutations and chromosomal abnormalities with known prognostic effect. The corresponding Kaplan-Meier estimates are illustrated in Figure S8 and S9.

**Table S10.** Median overall survival (OS) times for recurrent chromosomal abnormalities in AML and/or for mutations and chromosomal abnormalities with known prognostic effect. The corresponding Kaplan-Meier estimates are illustrated in Figure S8 and S9.

| Chromosomal abnormality |  | Median OS (Months) |
| --- | --- | --- |
| Complex karyotype | negative  positive | 8.3  6.1 |
| Monosomal karyotype | negative  positive | 8.1  4.8 |
| -7/del7q | negative  positive | 8.0  7.1 |
| -5/del5q | negative  positive | 7.7  7.0 |
| +8 | negative  positive | 7.9  6.2 |
| -17/del17p/abn(17p) | negative  positive | 7.5  6.9 |
| del(20q) | negative  positive | 7.7  6.2 |
| del(12p) | negative  positive | 7.5  8.7 |
| del(3p) | negative  positive | 7.6  5.7 |
| Monosomy 18 | negative  positive | 7.7  3.9 |

**SUPPLEMENTARY FIGURES**

**Figure S1**. Kaplan-Meier plots of overall and event-free survival of the 604 patients included in this study and randomized to guadecitabine (GUA) or treatment choice (TC; azacitidine, decitabine, low-dose cytarabine) within the ASTRAL-1 trial.

**Figure S2.** Lollipop plot displaying location and type of somatic and suspected germline mutations in *DDX41* detected in the ASTRAL-1 cohort (a). Co-mutational profile of *DDX41* mutated AML with a germline vs. somatic variant (b).

**a**

**b**

**Figure S3.** **Pairwise associations among genes mutated in ≥4% of AML (a), and pairwise associations between mutations and cytogenetic aberrations present ≥4% of the patients (b).**

Mutations and cytogenetic abnormalities were tested for mutual exclusivity and co-occurrence. After adjustment for multiple testing, 51 and 178 significant pairwise gene-gene and gene-cytogenetics associations, respectively, were detected. Mutations in *NPM1* which are commonly found in *de novo* AML showed mutual exclusivity with gene mutations associated with secondary AML (*ASXL1, RUNX1, BCOR*, *U2AF1)*, and *TP53*, but co-occurred with *FLT3*, *DNMT3A*, *IDH1*, *TET2, PTPN11* and *IDH2*^R140^ mutations. *TP53* was associated with adverse-risk cytogenetic abnormalities (complex or monosomal karyotype, -17/abn(17p), -5/del(5q), -7/del(7q)), but was mutually exclusive with *NPM1*, myelodysplasia-related gene mutations (*RUNX1*, *SRSF2*, *STAG2, BCOR*), *FLT3*, and mutations involved in clonal hematopoiesis (*DNMT3A*, *TET2*, *ASXL1*); overall, compared to AML with wildtype *TP53*, AML with mutated *TP53* had significantly less co-mutations (5 vs. 3; p<0.001). Further, myelodysplasia-related gene mutations (*ASXL1, RUNX1, SRSF2, STAG2)* were mutually exclusive with complex or monosomal karyotypes and other myelodysplasia-related cytogenetic abnormalities such as -17/abn(17p), -5/del(5q), -7/del(7q), del(3p). Mutations in the splicing factors *SRSF2* and *U2AF1* were mutually exclusive, as were genes involved in methylation such as *TET2* and *IDH2*^R140^ suggesting functional redundancy in the same biological pathway. However, *DNMT3A* showed a strong association with *IDH2*^R172^ (but not with *IDH2*^R140^) and *IDH1*.

Fisher’s exact test using a modified Benjamini-Hochberg procedure to control the false discovery rate (FDR) for discrete test statistics was used; blue colors illustrate co-occurrence, red colors illustrate mutual exclusivity; *p<.05, **p<.01, ***p<.001.

**a**

**b**


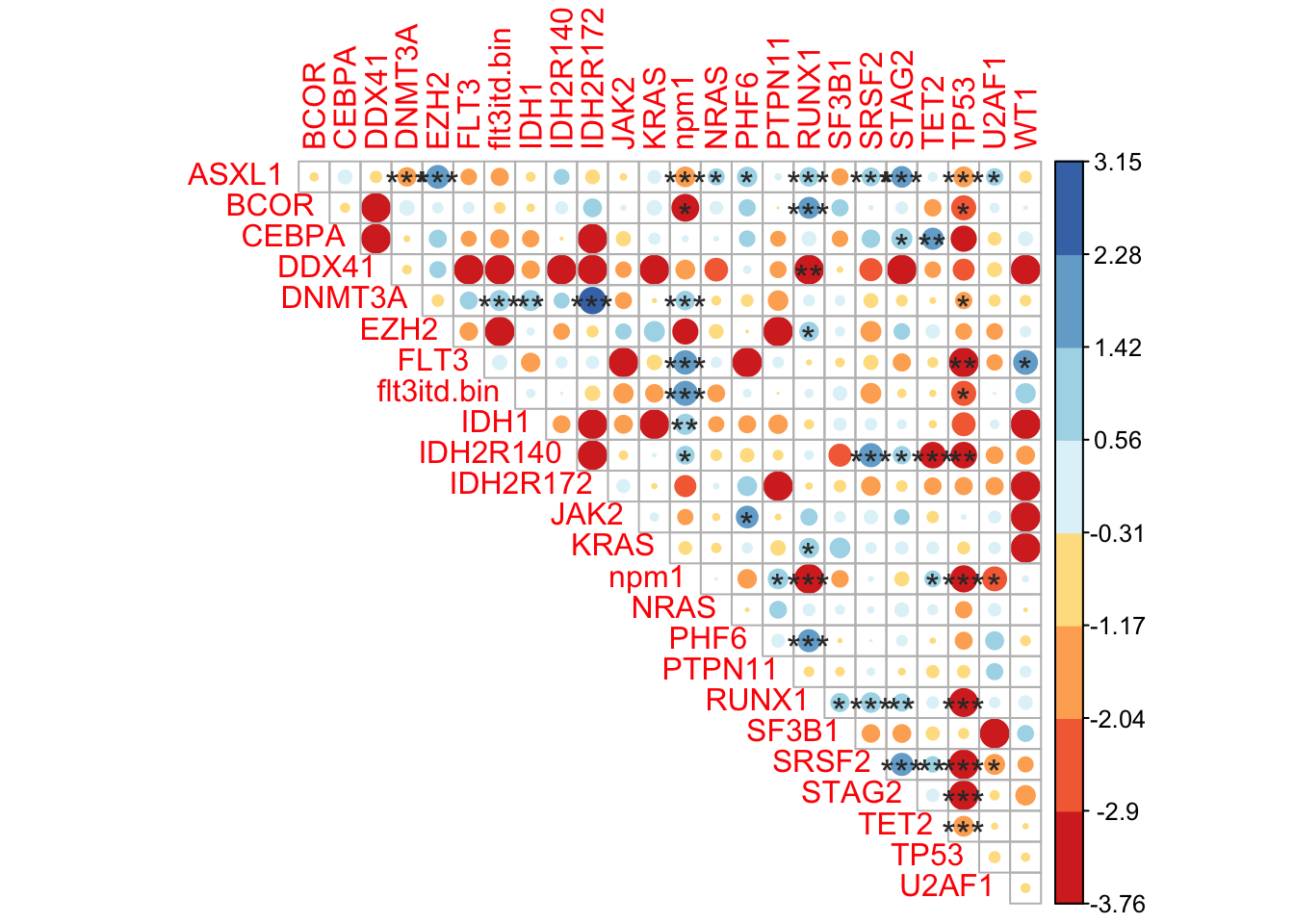


FLT3-ITD

FLT3-ITD

NPM1

NPM1

**Figure S4.** Bradley-Terry model of temporal acquisition of gene mutations using variant allele frequency (VAF). *DDX41* mutations, which were often germline, as well as mutations in genes such as *DNMT3A, TET2, JAK2*, and *TP53* that have been associated with clonal hematopoiesis of indeterminate potential (CHIP) occurred early during leukemogenesis suggesting disease initiating events, followed by mutations in chromatin-cohesin and splicing genes such as *U2AF1, SF3B1, SRSF2*, and *STAG2*. In line with previous reports, mutations in signaling genes such as *KRAS, NRAS, FLT3,* and *PTPN1*1 were late events, with the exception of *JAK2* mutations that arose early in disease development. A 4% threshold was used for mutation frequency, i.e., genes that were mutated in ≥24 patients were analyzed. The figure shows the VAF density plots ordered by Terry-Bradley estimates of temporal acquisition; VAFs corrected for X-chromosomal genes; not corrected for copy number alterations.


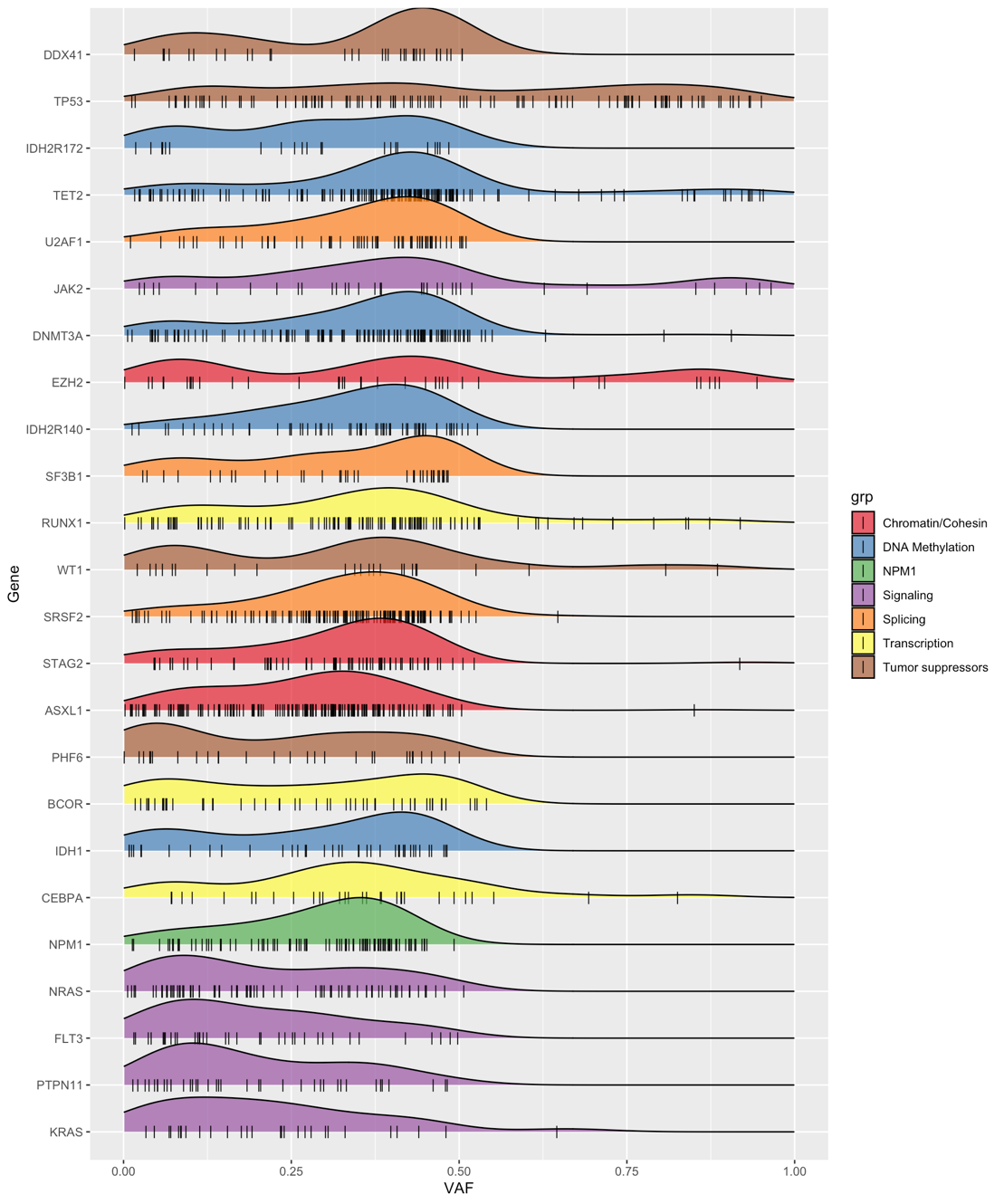


**Figure S5.** Impact of *DDX41* mutations on overall survival within the ICC categories “AML not otherwise specified” (NOS) (a) and “AML with myelodysplasia-related gene mutations” (b). Kaplan-Meier plots showing favorable prognosis in both ICC categories in the presence of *DDX41* alterations.

**a**

AML NOS


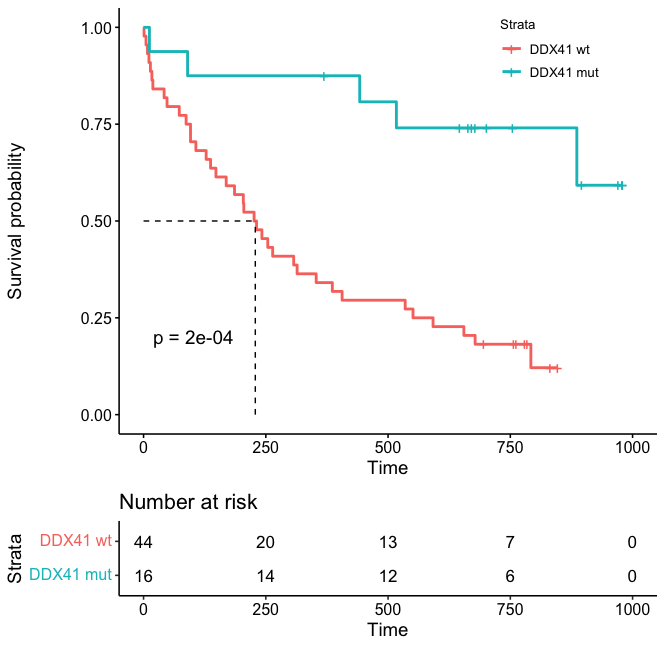


**b**

AML with myelodysplasia-related gene mutations


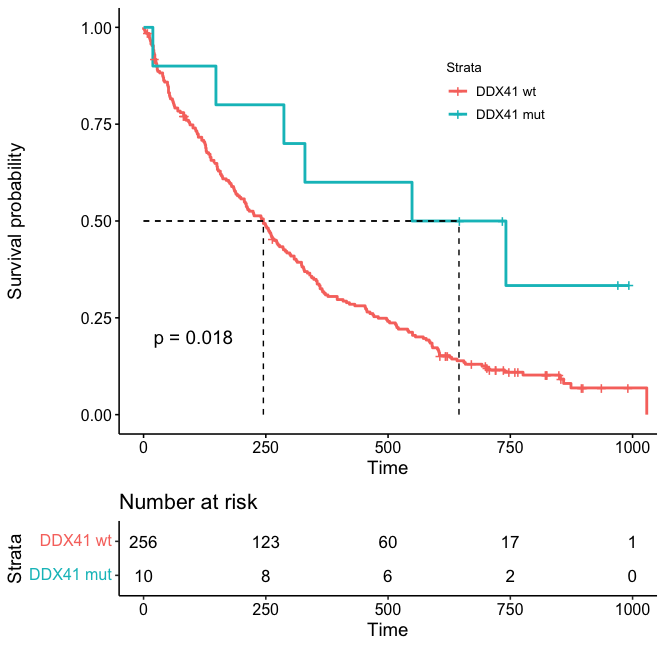


**Figure S6.** Forest plot displaying hazards ratios based on results from Cox regression analysis using clinical variables [age, sex, ECOG performance status (≥2 vs. <2), white blood cell counts (log10-transformed)], treatment, gene mutations with a frequency of ≥4%, as well as complex karyotype and cytogenetic abnormalities with a frequency of ≥4%. HR = hazard ratio, CI = confidence interval.

**Male Sex**

**Treatment Arm**

**FLT3-ITD**

**ECOG ≥ 2**

**Complex karyotype**

**-5/del(5q)**

**del(7q)**

**del(12p)**

**del(20q)**

**-17/del(17p)/abn(17p)**

**del(3p)**

**-7**

**+8**

**-18**

**-21**

**p-value**

**HR**

**(CI range)**

**Figure S7.** Cox regression analysis from Figure S6 now displaying values for variance inflation factor (VIF) of variables in the model to measure the amount of multicollinearity. High VIF values were calculated for the variables *TP53*, complex karyotype, and -5/del5q suggesting a strong correlation among the three variables.

del(3p)

-7

+8

-18

-21

-5/del(5q)

del(7q)

del(12p)

del(20q)

-17/del(17p)/abn(17p)

**Figure S8.** Impact of selected gene mutations on overall survival. Kaplan-Maier plots of overall survival using univariate analysis: *DDX41, TP53, FLT3-*ITD*, NPM1, DNMT3A, TET2,* myelodysplasia-related genes (*ASXL1, BCOR, EZH2, RUNX1, SF3B1, SRSF2, STAG2, U2AF1, ZRSR2), IDH1, IDH2, NRAS, KRAS, FLT3*-TKD, *PTPN11*. Univariate analysis was performed for recurrent mutations in AML and/or for mutations with known prognostic effect. Time is displayed in months. The corresponding 1-, 2-year, and median survival is displayed in Table S7 and S9.

**
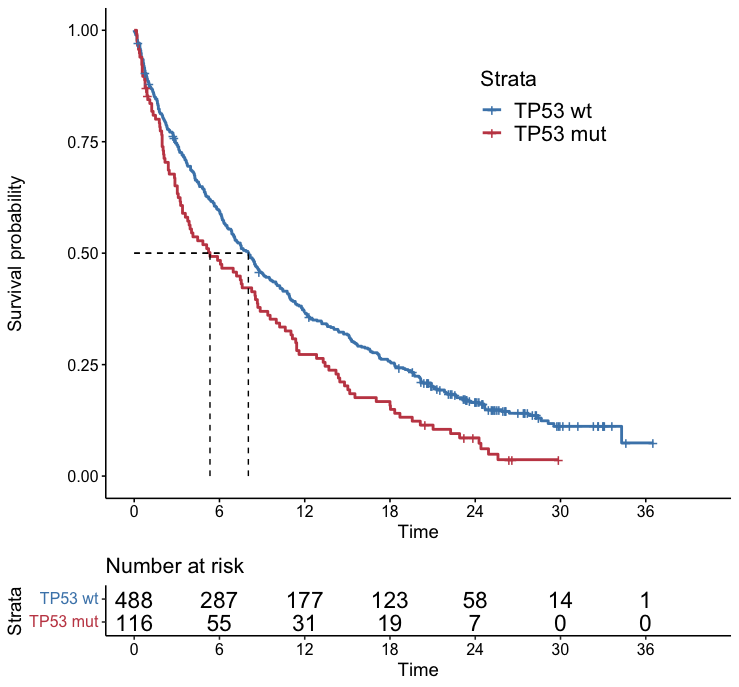
***
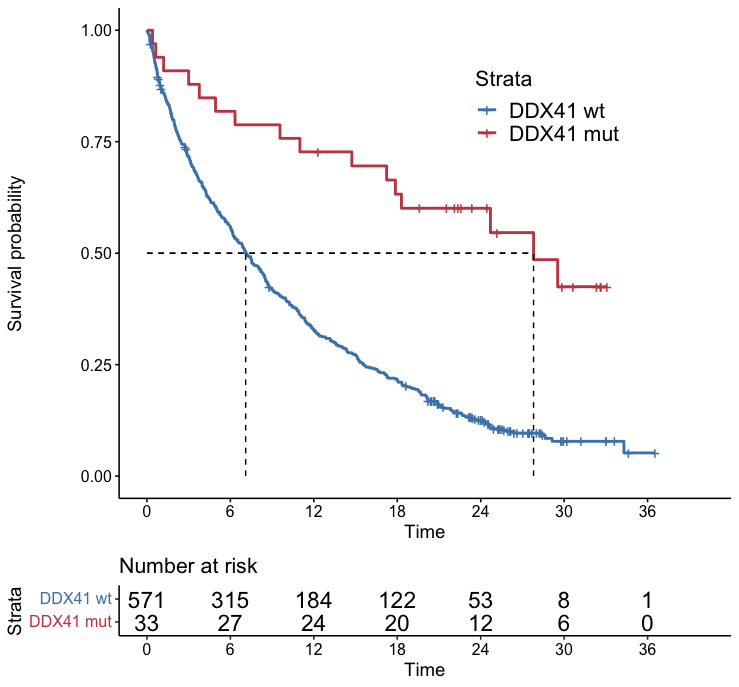
*

**
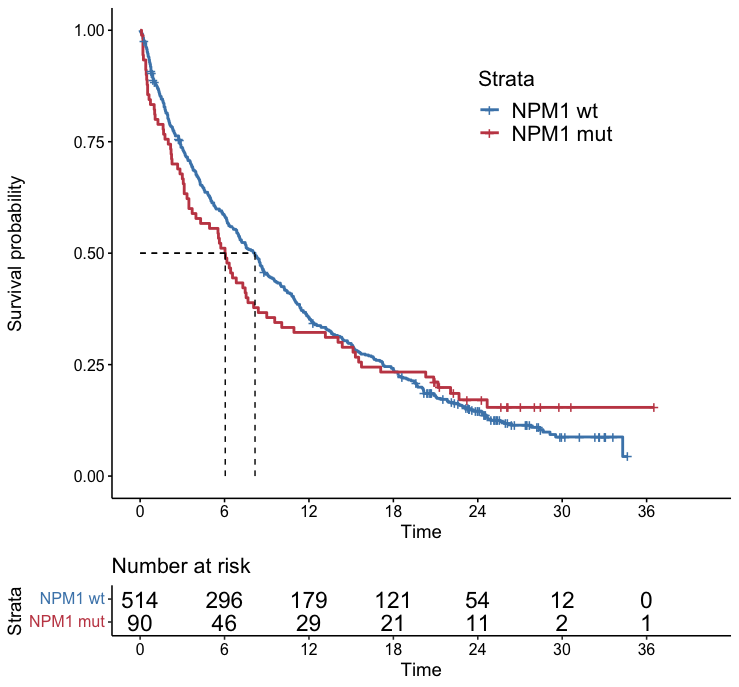

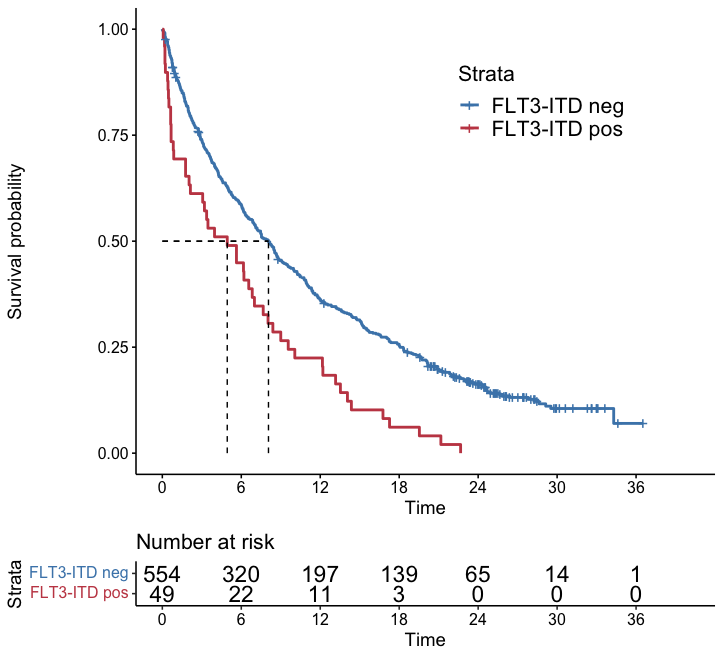
**

**
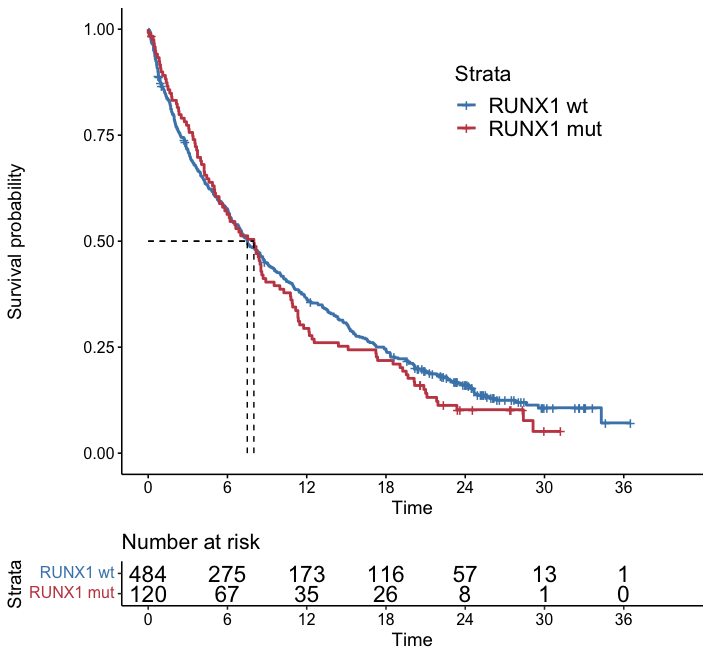

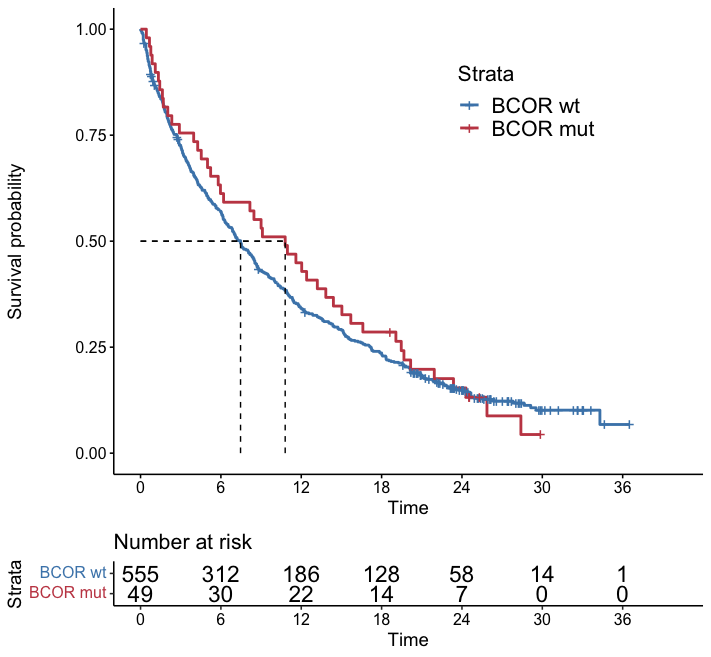

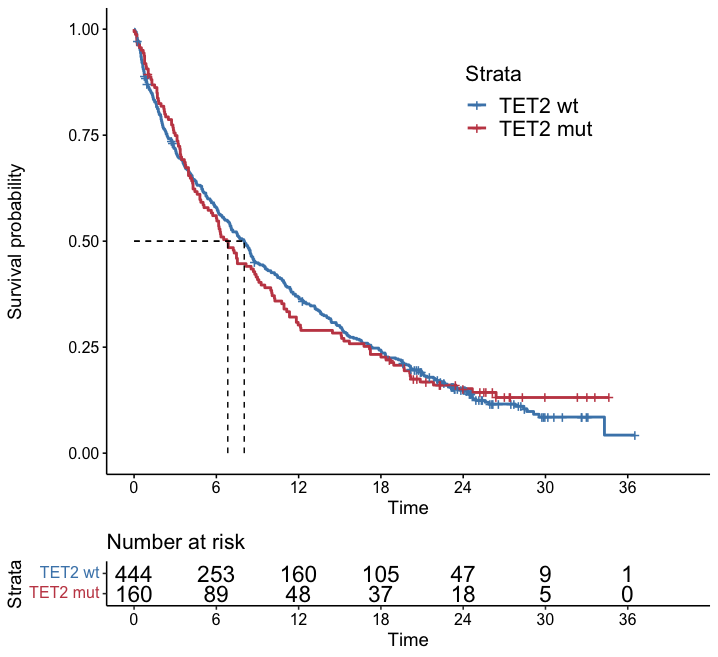

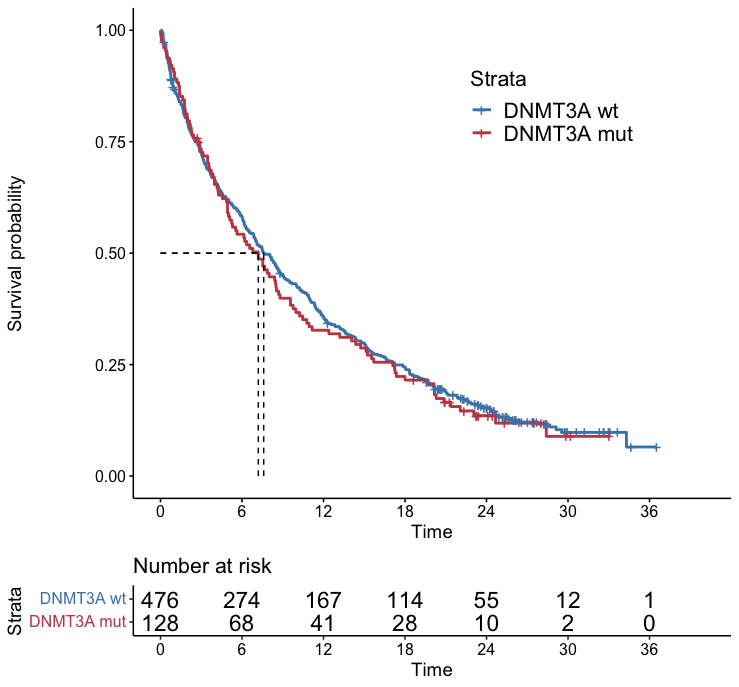

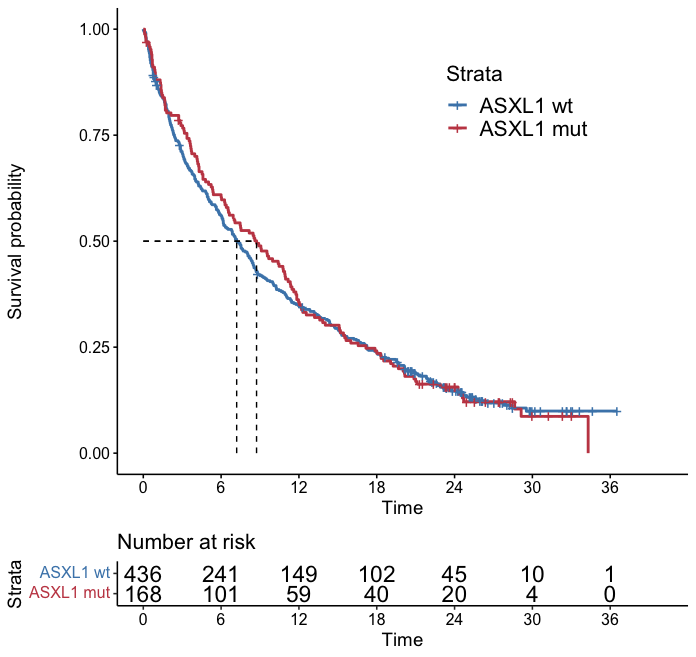

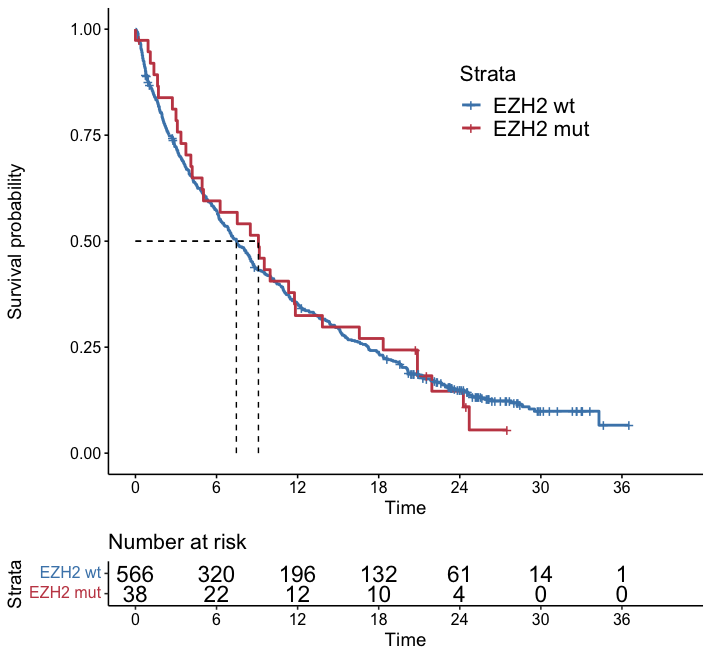
**


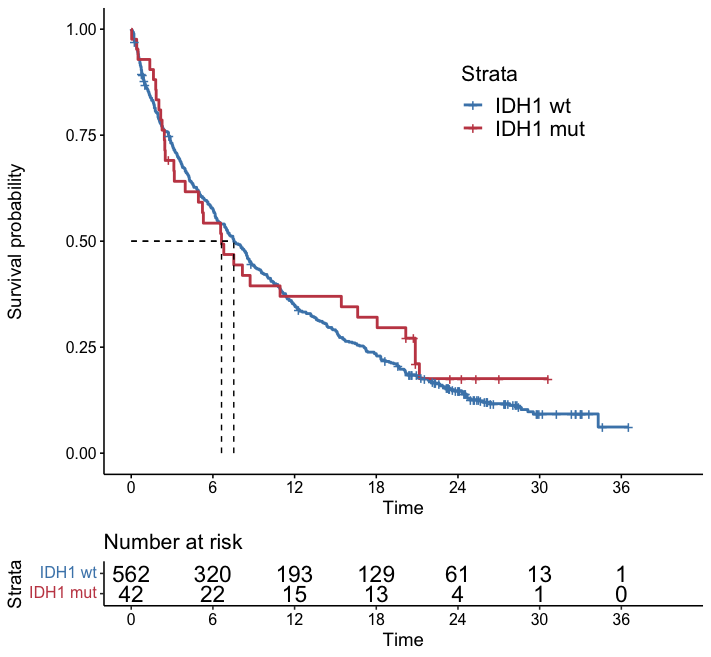
**
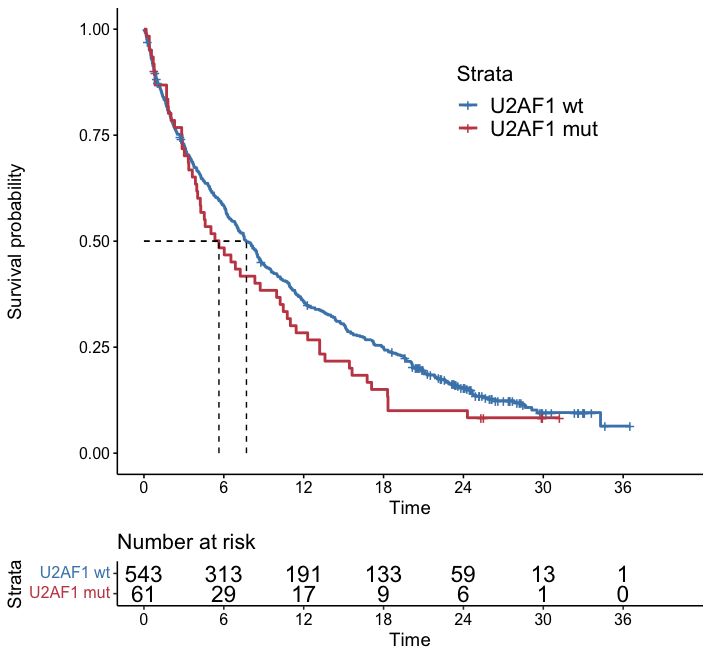
**
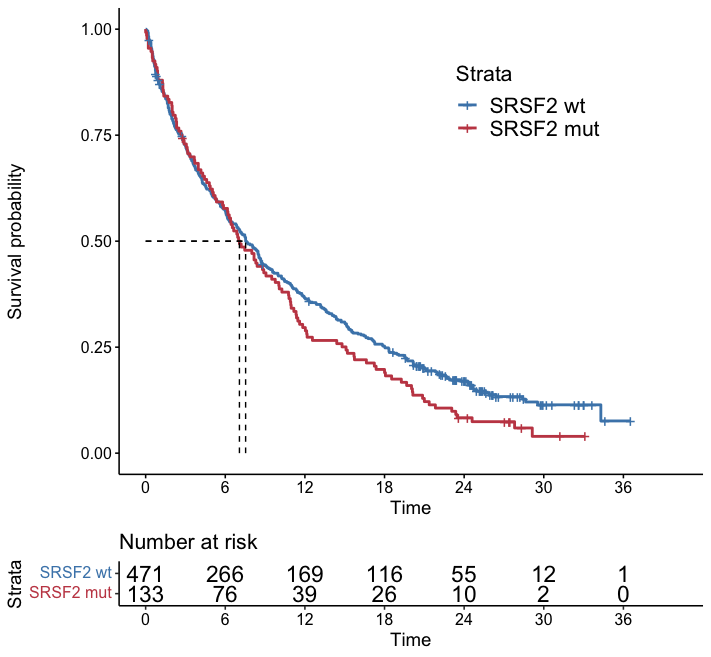
**
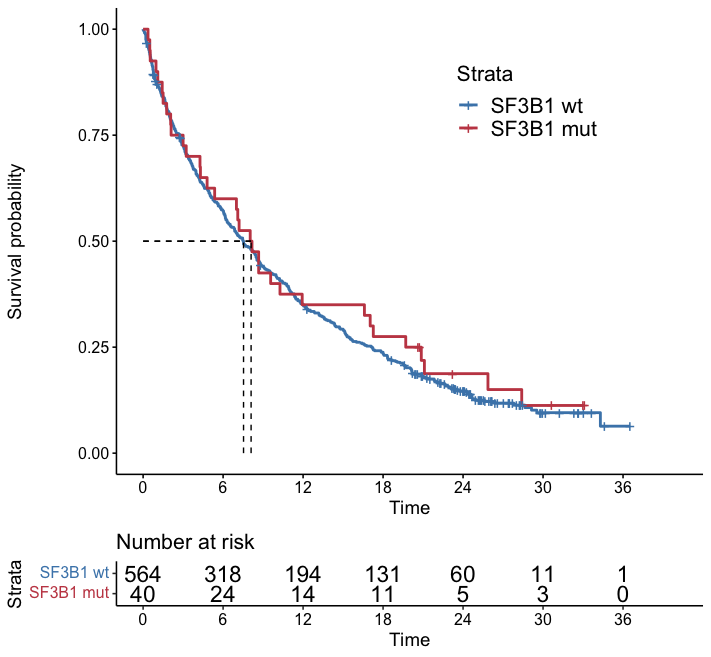

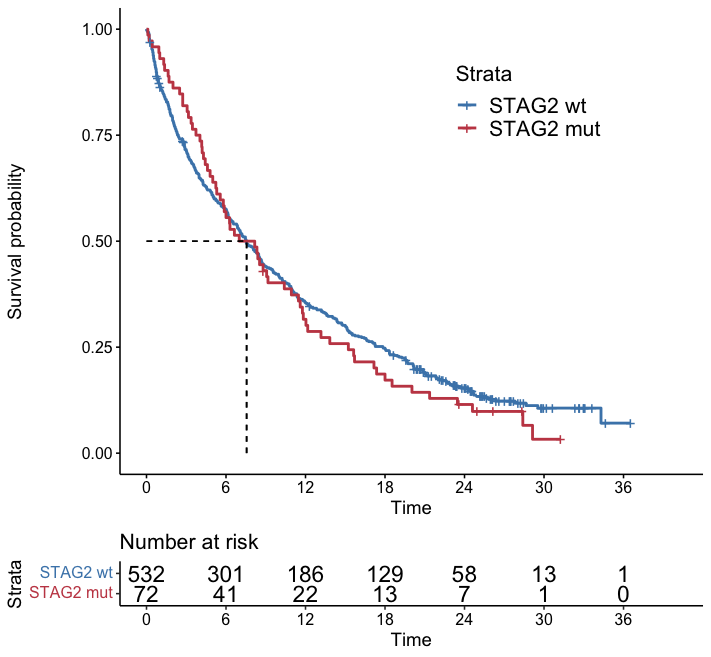
**
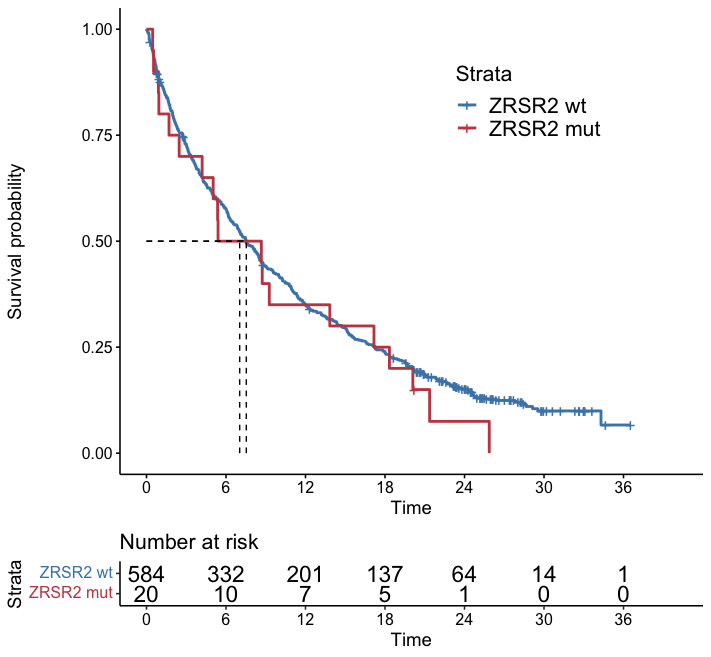
 **
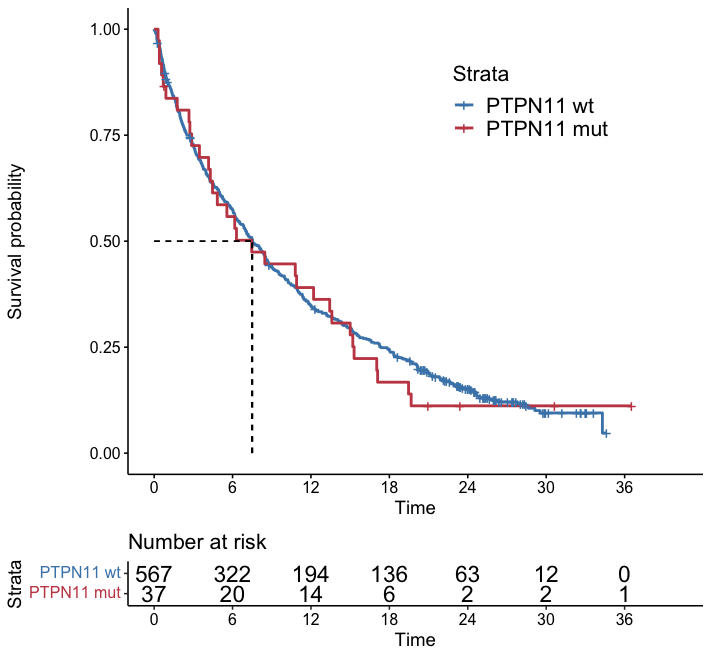

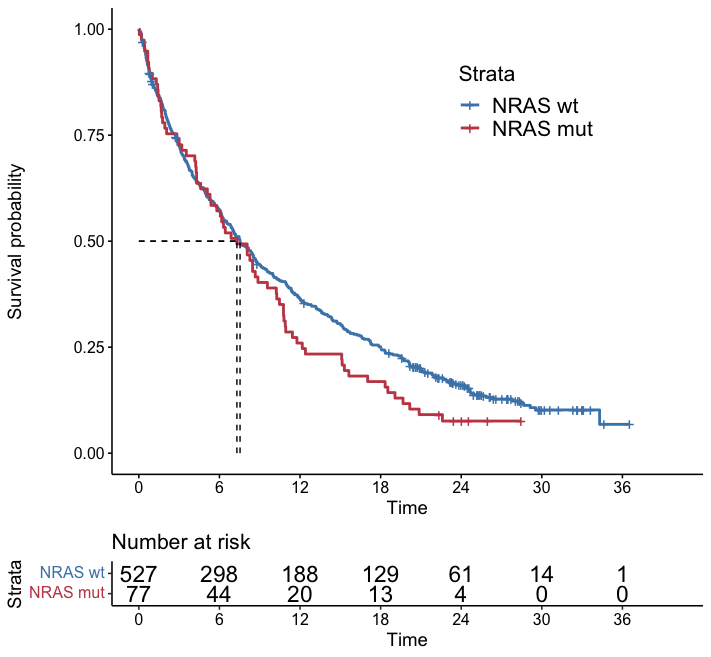

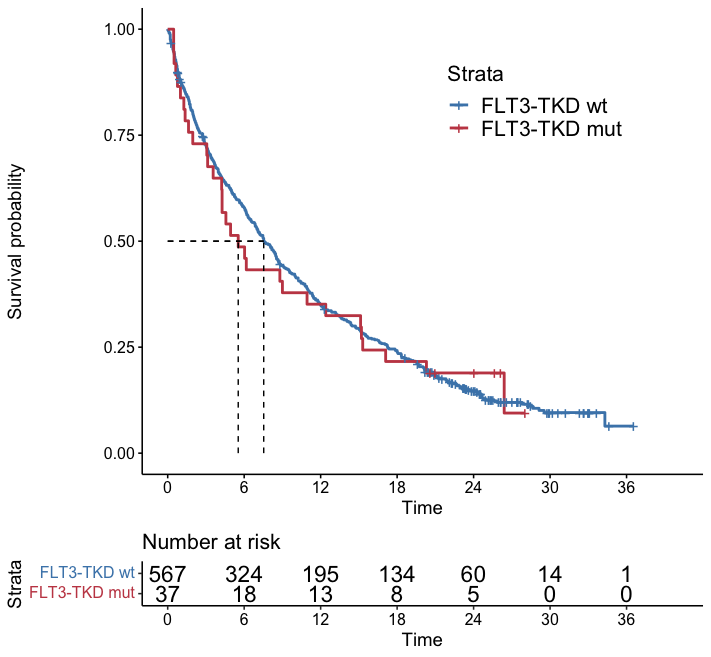

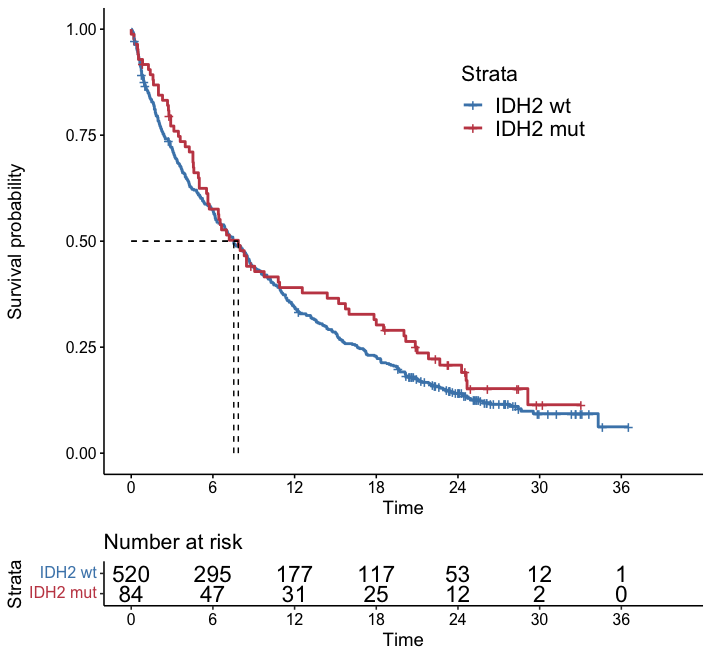

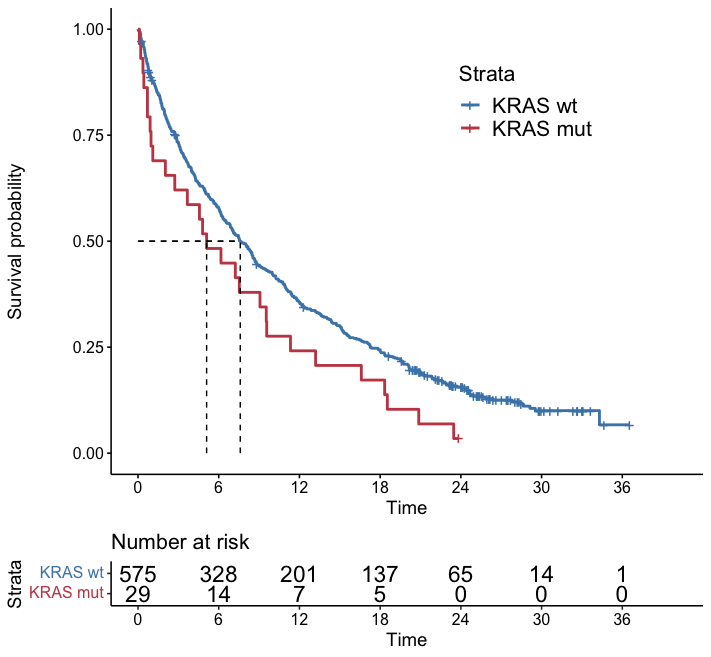
**

**Figure S9.** Impact of selected chromosome abnormalities on overall survival. Kaplan-Maier displaying the impact of cytogenetic abnormalities in univariate analysis such as complex karyotype, monosomal karyotype, -7/del(7q), -5/del(5q), 17/del(17p)/abn(17p), trisomy 8, del(3p), del(12p), del(20q), and monosomy 18. The univariate analysis was performed for recurrent aberrations in AML or aberrations with known prognostic effect. Time is displayed in months. The corresponding 1-, 2-year, and median survival is displayed in Table S8 and S10.


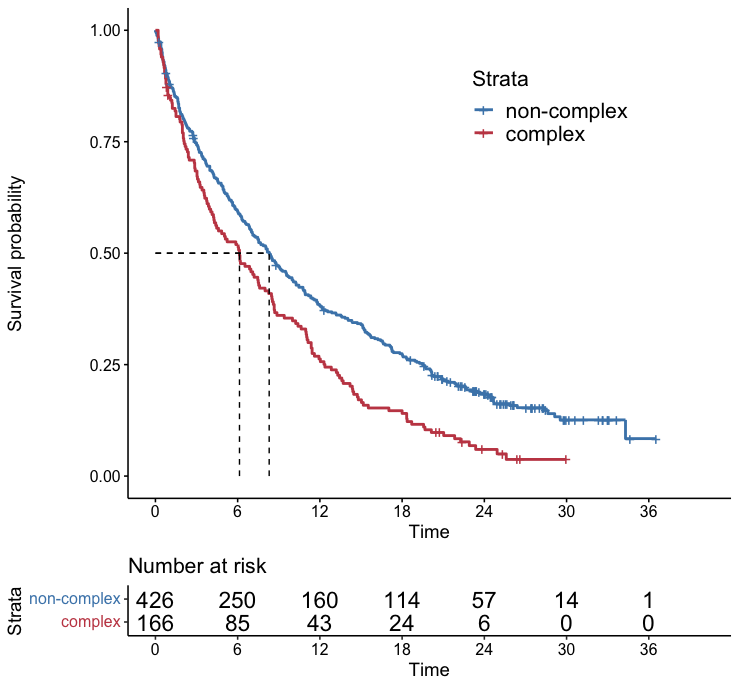
**
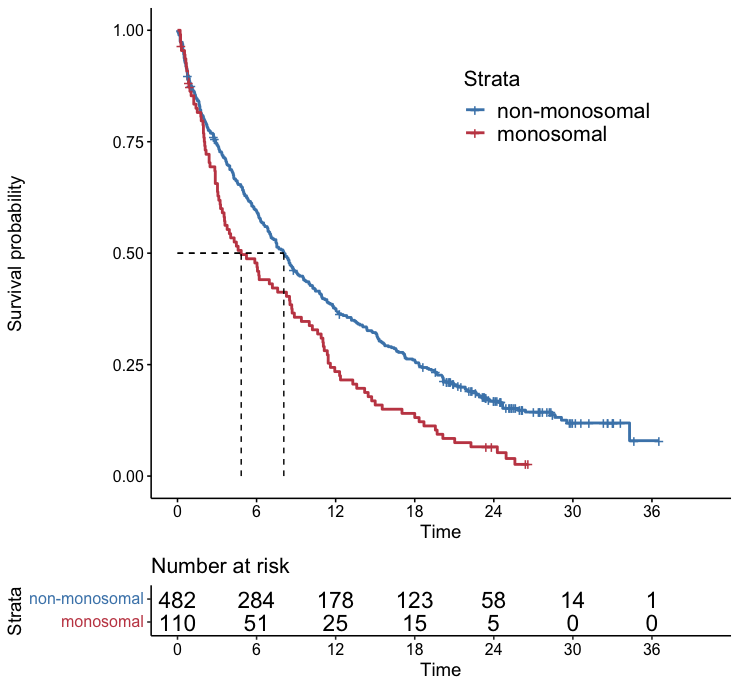

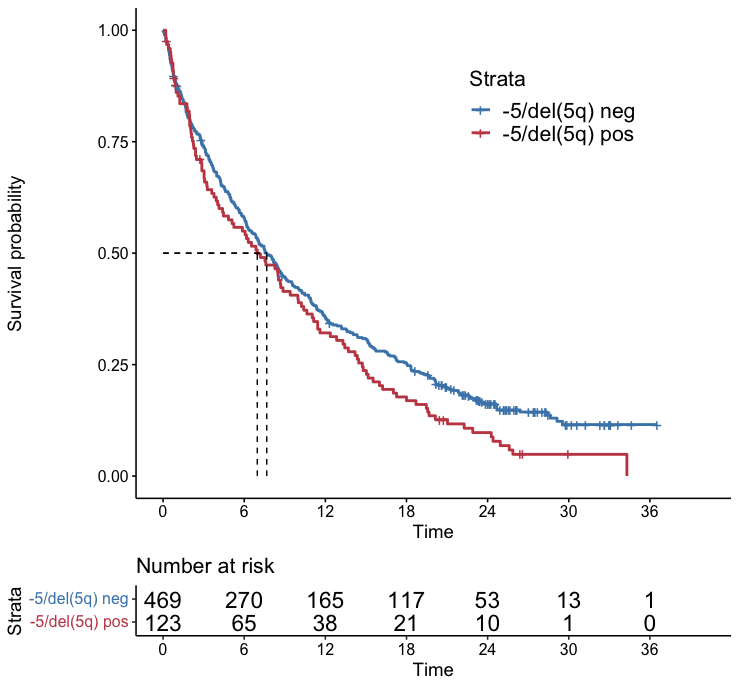
**

**
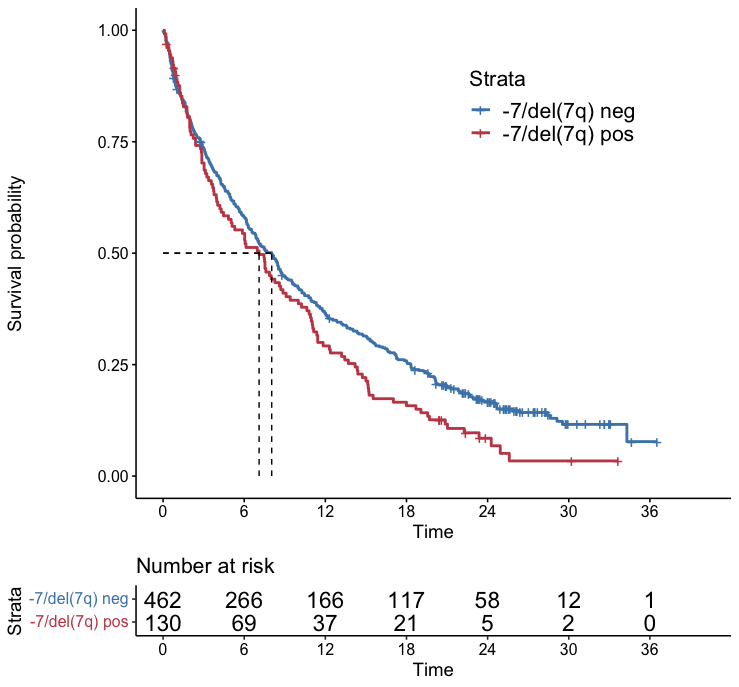
**

**
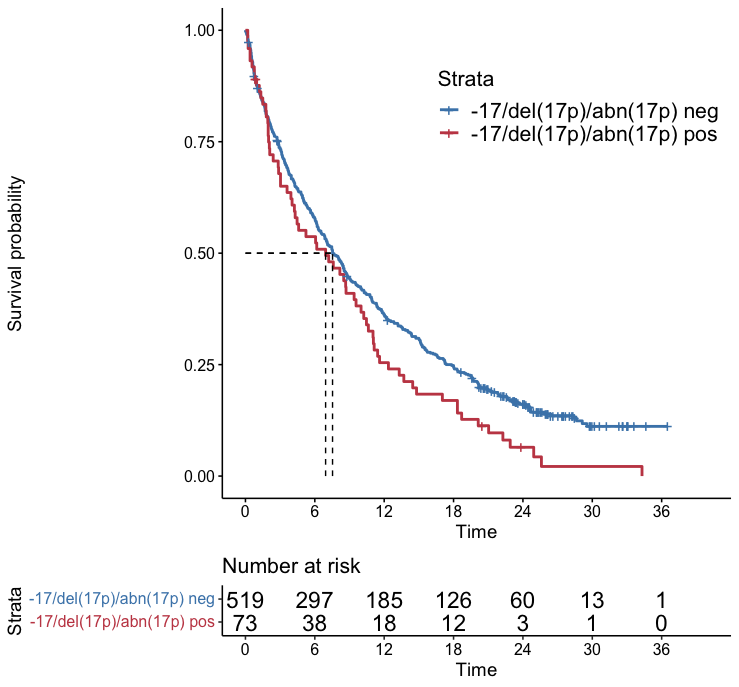

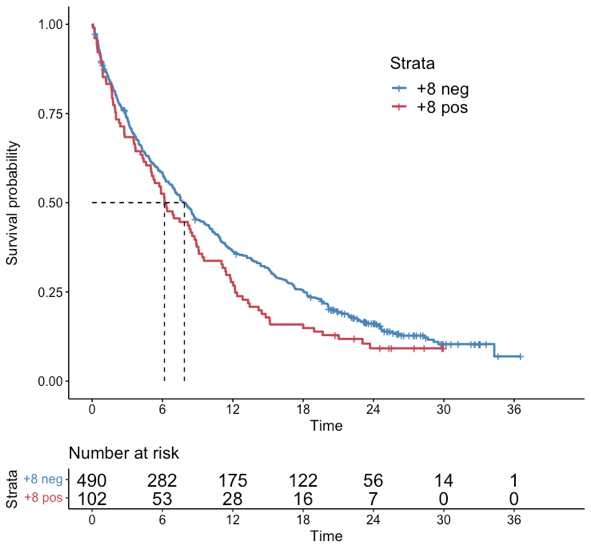
**

**
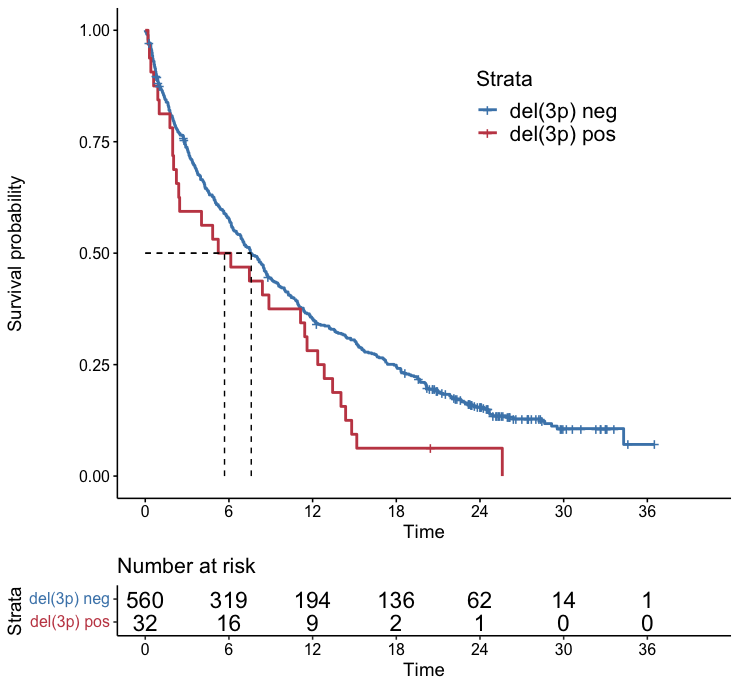

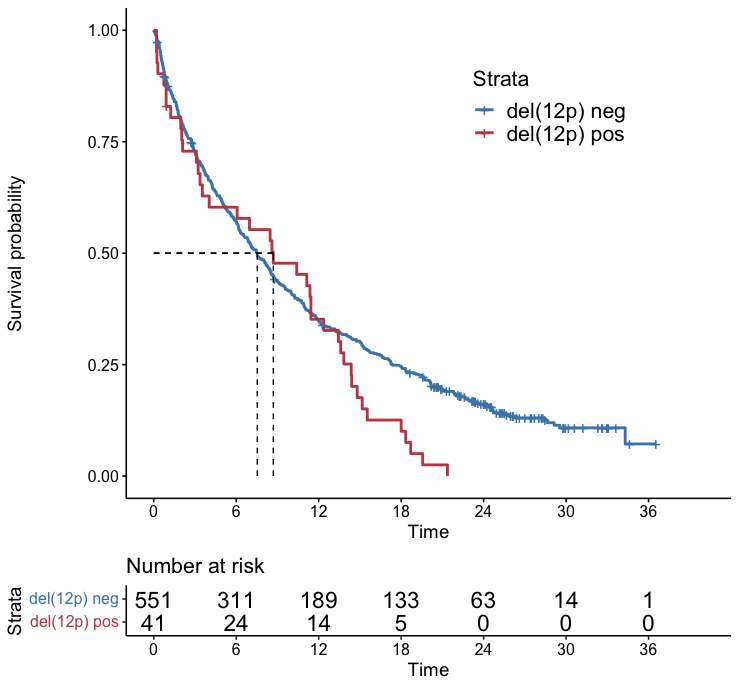
**

**
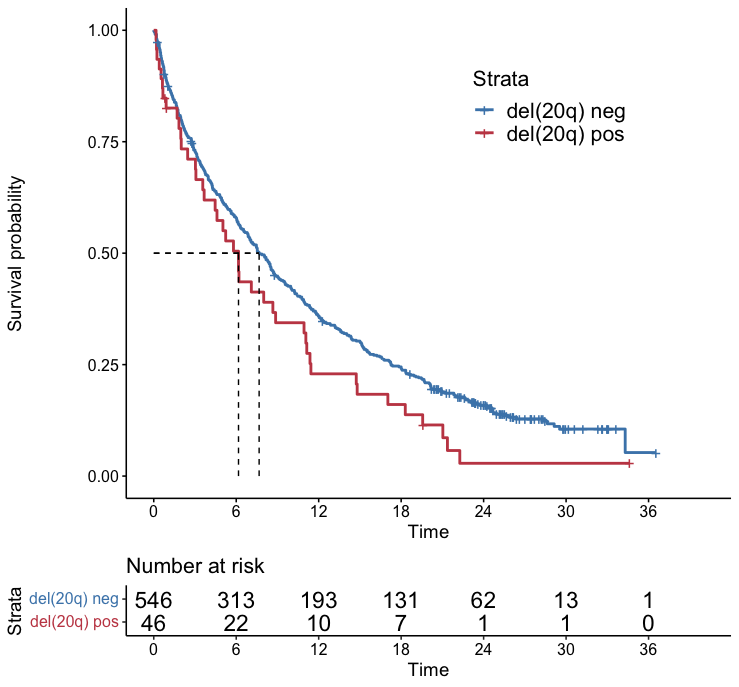

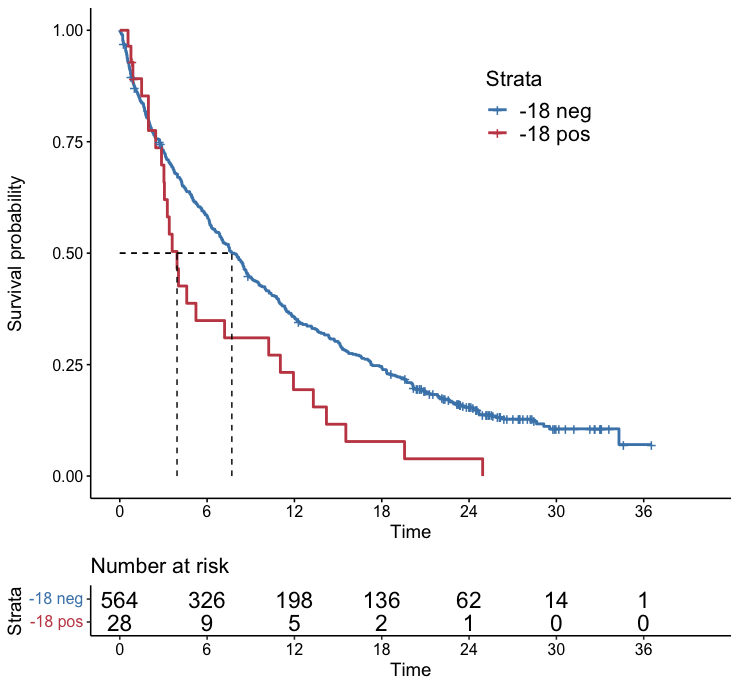
**

**Figure S10.** Values of integrated Brier score (crps). The prediction performance (in terms of the time-dependent Brier score) of the full model including five clinical variables (age, gender, WBC, ECOG and treatment arm) and all gene mutations with frequencies of at least 4% (Full) and the reduced model after backward selection (BackwardSel) including only ECOG, WBC, *FLT3*-ITD, *TP53,* and *DDX41* mutations was compared to a random survival forest (1000 trees) (RF) based on the same variables as the full model, a basic model including the five clinical variables only (Clinical) and the Kaplan-Meier estimate using no covariate information (Reference).

**Figure S11. Cluster plot illustrating the co-occurrence of mutational and cytogenetic alterations in cases with an NPM1 mutation (n=93).** Mutations present in $\geq$3% of cases are displayed. With regard to cytogenetics, associations with normal karyotype and adverse risk cytogenetic features (based on ELN 2022) are displayed.

Sixty-eight percent (n=63/93) of the cases had normal karyotype. Molecular features such as myelodysplasia-related gene mutations (45%, n=42/93) or FLT3-ITD (25%, n=23/93,) were common in AML with NPM1 mutation. DNMT3A mutations were detected in 40% (n=37/93) of the cases.
